# Structure-based development of new RAS-effector inhibitors from a combination of active and inactive RAS-binding compounds

**DOI:** 10.1073/pnas.1811360116

**Published:** 2019-01-25

**Authors:** Abimael Cruz-Migoni, Peter Canning, Camilo E. Quevedo, Carole J. R. Bataille, Nicolas Bery, Ami Miller, Angela J. Russell, Simon E. V. Phillips, Stephen B. Carr, Terence H. Rabbitts

**Affiliations:** ^a^Weatherall Institute of Molecular Medicine, MRC Molecular Haematology Unit, John Radcliffe Hospital, University of Oxford, OX3 9DS Oxford, United Kingdom;; ^b^Research Complex at Harwell, Rutherford Appleton Laboratory, OX11 0FA Didcot, United Kingdom;; ^c^Chemistry Research Laboratory, University of Oxford, OX1 3TA Oxford, United Kingdom;; ^d^Department of Biochemistry, University of Oxford, OX1 3QU Oxford, United Kingdom

**Keywords:** cancer, RAS, drugs, antibody, intracellular antibody

## Abstract

The *RAS* gene family is frequently mutated in human cancers, and the quest for compounds that bind to mutant RAS remains a major goal, as it also does for inhibitors of protein–protein interactions. We have refined crystallization conditions for KRAS_169_^Q61H^-yielding crystals suitable for soaking with compounds and exploited this to assess new RAS-binding compounds selected by screening a protein–protein interaction-focused compound library using surface plasmon resonance. Two compounds, referred to as PPIN-1 and PPIN-2, with related structures from 30 initial RAS binders showed binding to a pocket where compounds had been previously developed, including RAS effector protein–protein interaction inhibitors selected using an intracellular antibody fragment (called Abd compounds). Unlike the Abd series of RAS binders, PPIN-1 and PPIN-2 compounds were not competed by the inhibitory anti-RAS intracellular antibody fragment and did not show any RAS-effector inhibition properties. By fusing the common, anchoring part from the two new compounds with the inhibitory substituents of the Abd series, we have created a set of compounds that inhibit RAS-effector interactions with increased potency. These fused compounds add to the growing catalog of RAS protein–protein inhibitors and show that building a chemical series by crossing over two chemical series is a strategy to create RAS-binding small molecules.

## Significance

The RAS family of oncogenic proteins is important as therapy targets because of the frequency of activating mutations in almost all major cancers. An important approach is development of small molecules with drug-like properties that can inhibit RAS-effector protein interactions inside cells. We present a strategy for identification of such compounds, and their development as RAS-effector interaction inhibitors, utilizing a structure-based design approach and cell-based assays. By combining moieties from two distinct sets of RAS-binding molecules, we generated cross-over compounds that showed improved efficacy in vitro and in cell-based assays.

The oncogenic family of *RAS* genes is of significant interest in the fight against cancer because of the frequency of activating mutations (1). Their presence in almost all major cancers makes them a highly valued therapeutic target, in particular the KRAS gene, since it has been identified as one of the most frequently mutated oncogenes (2, 3). RAS proteins are linked to the plasma membrane by COOH-terminal prenylation mediated by farnesyl transferases (4). All family members function by signal transduction to the nucleus of cells via interaction with effectors (such as RAF, RALGDS, and PI3K) that catalyze phosphorylation of downstream proteins (5). When KRAS is bound to GDP, the protein is in the inactive state and becomes activated by nucleotide exchange from GDP to GTP. Normally, the activation/deactivation cycle is catalyzed by guanine nucleotide exchange factors and GTPase-activating proteins (GAPs) (6, 7). Mutant RAS proteins remain in the active state and hydrolyze GTP at a much slower rate than wild-type (WT) RAS (8). Mutations reduce GAP activity leading to constitutive activation of RAS effector pathways (2), constantly generating a signaling cascade that activates cell functions such as division, survival, and invasion (9).

Despite its great potential as a cancer target, KRAS has proved to be very difficult to inhibit in a therapeutic setting. KRAS signaling works via protein–protein interactions (PPI) that can be very difficult to disrupt (10). In addition, the nucleotides that regulate KRAS function (GTP and GDP) bind to the protein with picomolar affinity, making them problematic to displace (11). Attempts at targeting RAS function using farnesyl transferase inhibitors also proved to be ineffective, failing to demonstrate antitumor activity in KRAS-driven cancers (12). As an alternative to compounds, various macromolecules [called macrodrugs (13)] have been developed that can bind to RAS and prevent PPI with the RAS effectors, such as has been shown with intracellular antibody fragments (14, 15). The possible clinical use of these macrodrugs has not been implemented thus far due to difficulties in their delivery into cells, although methods are becoming available that may solve this problem (16).

Although there are a large number of mutant RAS protein isoforms, their structural conformation is highly conserved (17) because of the invariant N-terminal domain up to amino acid 166. The interest in inhibition of RAS proteins by small molecules has increased again recently (18), and several compounds have been described that bind to RAS (19–27). Recently, we have defined a chemical series based on an intracellular antibody-binding domain (28) that interact with a hydrophobic pocket (designated pocket I, *SI Appendix*, Fig. S1*A*), previously identified in silico (29) and confirmed as the binding site for 4,6-dichloro-2-methyl-3-aminoethyl-indole (DCAI) near the switch I region of KRAS (23).

A critical step in drug development programs for progressing small molecules is the use of X-ray crystallography with compounds after crystal soaking or cocrystallization to identify where such molecules bind to the target protein. We have optimized KRAS_169_^Q61H^ crystallization and applied crystal soaking to assess a set of RAS-binding compounds selected from an initial diverse PPI-net l compound library (kindly provided by Andrew Wilson, University of Leeds, Leeds, UK), of which two bind in pocket I. However, unlike our previous Abd compounds, their binding was not impaired by binding of an inhibitory anti-RAS intracellular antibody fragment nor did they interfere with RAS-effector interactions. Comparison of the structures of these two PPI-net RAS-binding compounds and the lead compound Abd-7 allowed us to synthesize chimeric cross-over compounds that bind to RAS with improved potency and inhibit RAS-effector interactions whereas the PPI-net did not.

## Results

### Crystallography Conditions for KRAS_169_^Q61H^ GppNHp, Suitable for Crystal Soaking.

**Fig. 1.**
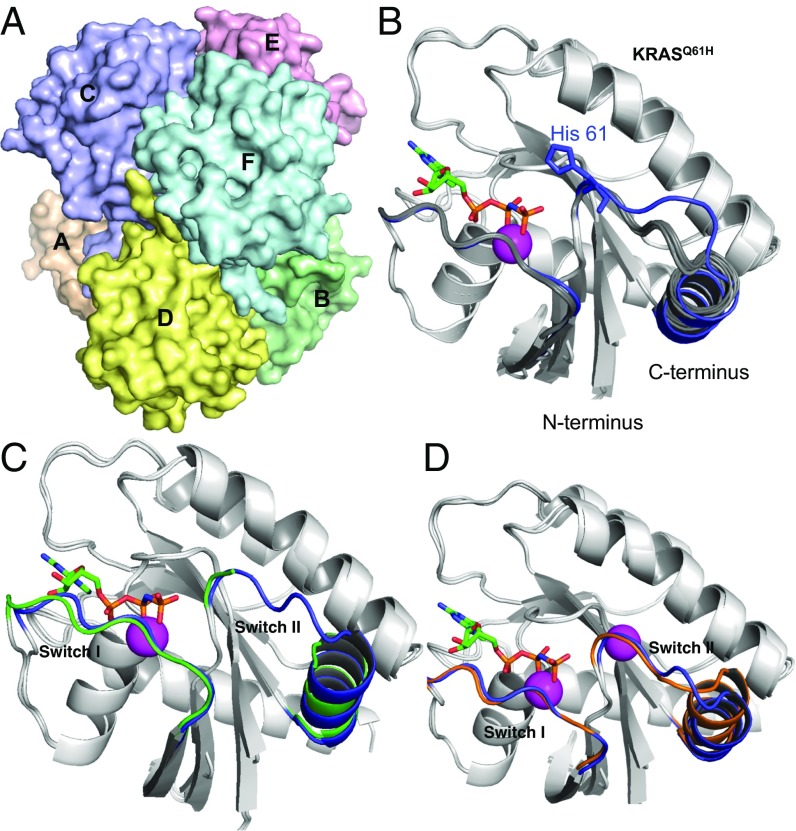
KRAS_169_^Q61H^ structure analysis using new crystallization conditions. KRAS_169_^Q61H^ protein was crystallized with bound GTP-analog GppNHp. (*A*) Surface representation of the asymmetric unit containing the six KRAS_169_^Q61H^ proteins in different colors with different chains, labeled A–F. (*B*) Ribbon representation of KRAS_169_^Q61H^ showing an overlay of the six chains, of the asymmetric unit. The switch regions of five proteins (B–F) are identical (depicted in dark gray), and one (chain A) has a stabilized switch I and switch II (depicted in blue) due to interactions with neighboring protein molecules in the crystal lattice. Residue H61 and GppNHp are indicated and one Mg atom (shown as a magenta sphere) was identified per chain. *C* and *D* show ribbon representation overlays of the KRAS_169_^Q61H^ (chain A) structure with KRAS_188_^G12V^ (*C*, switch I and switch II depicted in green) and KRAS_188_^G12D^ (*D*, switch I and switch II depicted in brown), highlighting structural conservation across RAS mutations.

Initially, we wanted to establish crystallization conditions to obtain a crystal form that would allow free movement of RAS-binding compounds through the lattice for crystal soaking. We reproduced the KRAS_169_^Q61H^ crystal structure found in the database (PDB ID code 3GFT) but with optimized crystallization conditions, using sparse matrix crystallization screening with a protein spanning residues 1–169. This produced diffraction quality crystals that showed similar packing to PBD ID code 3GFT with six chains in the asymmetric unit (Fig. 1*A* and *SI Appendix*, Table S1). All six crystallographically independent chains (identified as chains A–F) have the same fold. Noncrystallographic symmetry averaging of the electron density maps allowed the assignment of all of the polypeptide backbone for switch I (in all six chains). Switch II is more flexible in this crystal form and a complete model could only be built in three or four copies of RAS per asymmetric unit. Further evidence for the flexibility of switch II is that chain A adopted a different conformation in this region owing to interactions with a symmetry-related molecule (Fig. 1*B*). The six chains are less sterically hindered than in other published RAS crystal structures, such as KRAS_188_^G13D^ (21) PDB ID code 4DST. The solvent channels are also much larger, facilitating compound diffusion; consequently, bound compounds have more freedom to be accommodated within KRAS_169_^Q61H^ crystals, and the structures are more likely to represent the interaction in solution. In addition, six independent protein chains are available for binding of compounds within the asymmetric unit, making this crystal form particularly suitable for compound-soaking experiments.

As a comparison with the KRAS_169_^Q61H^ crystals, we also determined crystal structures of WT KRAS_188_, mutant KRAS_188_^G12V^ (*SI Appendix*, Fig. S2 and Table S2), and KRAS_188_^G12D^ (all isoform 4B) (*SI Appendix*, Fig. S3 and Table S2) with a GTP analog (GppNHp), using crystallization conditions similar to those described previously for full-length KRAS_188_^G12D^ (23). When the crystal packings of KRAS_169_^Q61H^ and KRAS_188_^G12D^ mutant proteins are compared, the switch regions are more solvent-accessible and less sterically hindered for the Q61H crystal. A further comparison was carried out between the switch regions of the two full-length KRAS structures (G12V and G12D) with our new KRAS_169_^Q61H^ structure (amino acids 1–169, Fig. 1 *C* and *D*). Both KRAS_169_^Q61H^ and KRAS_188_^G12V^ lack stabilization in the switch II region, and this instability could be attributed to the lack of a Mg ion binding to the switch II. All of the comparisons and observations between different crystal forms and mutants led us to conclude that the KRAS_169_^Q61H^ crystals were the best option for crystal-soaking experiments.

### PPI-Net Fragment Screen with KRAS_166_^G12V^.

**Fig. 2.**
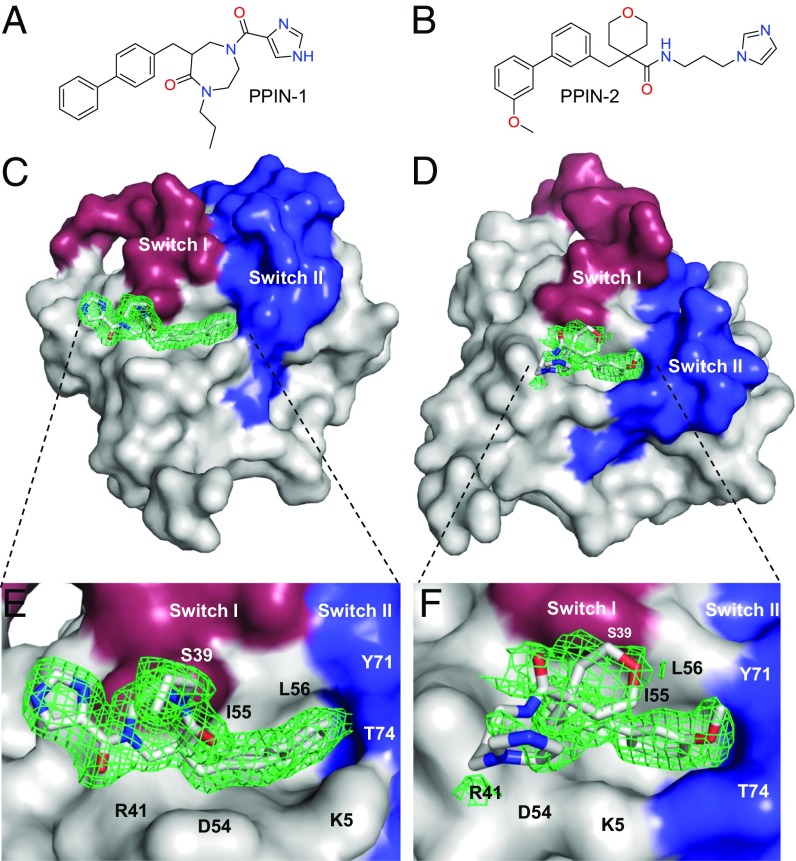
Crystal structure of PPIN-1 and PPIN-2 bound to KRAS_169_^Q61H^-GppNHp. The crystal structure of KRAS_169_^Q61H^ with PPIN-1 and PPIN-2 was derived by crystal soaking with the compounds (their structures are shown in *A* and *B*, respectively). (*C* and *D*) Surface representations of the binding of PPIN-1 and PPIN-2 into pocket I close to the switch regions I (red) and II (blue). Good 2mFo-DFc electron density (green mesh) was found for the whole of PPIN-1 and for the biphenyl head group of PPIN-2 (green mesh) but less contiguous for the rest of the molecule. (*E* and *F*) Expanded views of the interactions of PPIN-1 and PPIN-2 with KRAS with the following residues in contact: K5, L6, V7, S39, Y40, R41, D54, I55, L56, Y71, and T74.

We previously identified a compound series that binds to RAS in pocket I using a high-affinity anti-RAS intracellular antibody fragment in competition surface plasmon resonance (SPR) (28). In the present paper, we used direct screening of KRAS with a compound library using SPR. The library used was triaged for possible PPI inhibitors and comprised 1,534 compounds (the PP1-net screening collection). To identify specific KRAS binders, the library was simultaneously negatively screened against two control proteins, namely the LIM-only protein 2 (LMO2) and a fusion protein consisting of LMO2 bound by an intracellular antibody VH fragment (LMO2-VH fusion) (30). Responses were referenced by subtracting those measured against the control protein LMO2 from the responses measured against KRAS (R_ref_). Compounds were selected as hits if R_ref_ was over 10 RU and if compounds did not bind the LMO2-VH fusion protein. Thirty compounds bound to KRAS_166_^G12V^ (*SI Appendix*, Fig. S4*A*) of which 7 showed RAS specificity. Four of these compounds were available in sufficient quantities (with identities confirmed by mass spectroscopy) to allow waterLOGSY NMR to be carried out showing that PPIN-1 and PPIN-2 (the chemical structures are shown in Fig. 2 *A* and *B*) have good interaction properties with KRAS_166_^G12V^-GppNHp (*SI Appendix*, Fig. S4 *A*–*C*). Furthermore, the two PPIN compounds still bind to KRAS in waterLOGSY experiments in the presence of the RAS-inhibitory single-chain variable region antibody fragment (scFv) as a competitor. Thus, in this orthogonal assay, PPIN-1 and -2 were confirmed to bind to KRAS_166_^G12V^, but neither compound was prevented from binding to KRAS by the anti-RAS scFv intracellular antibody fragment as shown using Bioluminescence Resonance Energy Transfer (BRET)-based RAS biosensors (31) to assess intracellular RAS-protein interactions. As predicted from the waterLOGSY data using the intracellular antibody fragment, the PPIN compounds did not disrupt the interaction of KRAS_166_^G12D^ with the anti-RAS iDAb (VHY6) with a dematured version of the iDAb (VHY6dm) or with full-length CRAF (CRAF^FL^) (*SI Appendix*, Fig. S4*D*). This discrepancy between in vitro affinity and in cell potency could be attributed to targets with high conformational variability (like RAS switch regions) interacting with allosteric binders (32, 33).

### Crystal Soaking of KRAS_169_Q61H with PPIN-1 and -2.

We determined the RAS-binding sites of PPIN-1 and PPIN-2 by soaking KRAS_169_^Q61H^ crystals. Crystals of KRAS soaked with PPIN-1 diffracted to 1.63 Å (Fig. 2 *C* and *E* and *SI Appendix*, Table S3). Good electron density was observed for the ligand in one chain of the asymmetric unit (chain A, in which the switch regions are stabilized due to additional interactions on the opposite face with residues Arg102 and Lys101 from chain B in a neighboring asymmetric unit) and located adjacent to the C-terminal end of the switch I and switch II regions in pocket I. PPIN-1 primarily contacts KRAS_169_^Q61H^ with the biphenyl head group via van der Waals interactions. No hydrogen bonds are formed with the protein. Crystals soaked with PPIN-2 diffracted to 1.7 Å. For this compound, clear electron density corresponding to the methoxy-biphenyl anchor group was observed in four of the six chains (Fig. 2 *D* and *F*). Weaker electron density was observed around the linker and tail groups, suggesting that the rest of the compound remains flexible when bound to the protein. It was noteworthy that both PPIN compounds have the same biphenyl anchoring group but different tail functional groups, suggesting that the biphenyl-type groups of PPIN-1 and -2 are key in targeting these compounds to the pocket I-binding site.

### Design and Characterization of RAS-Binding Cross-Over Compounds.

**Fig. 3.**
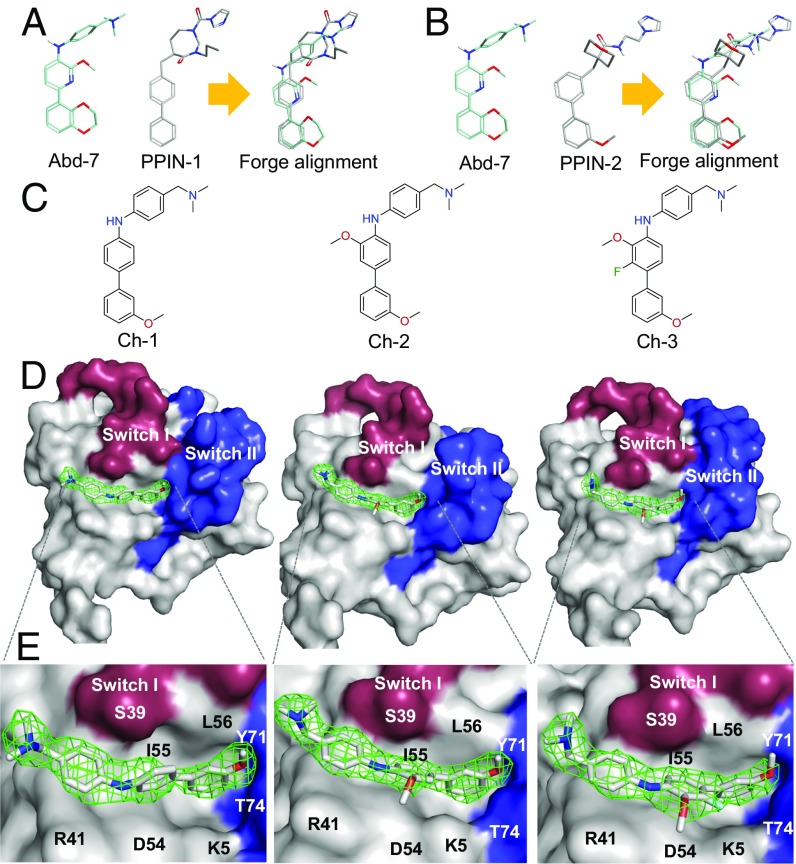
Abd and PPI-net compound alignment and cross-over compound crystallography. Alignments were carried out with the computational chemistry suite FORGE. (*A*) Abd-7 and PPIN-1 with alignments. (*B*) Abd-7 and PPIN-2. Three cross-over compounds were synthesized after the alignments, which are shown in *C* (*Left*, Ch-1; *Middle*, Ch-2; *Right* Ch-3). These compounds were soaked into KRAS_169_^Q61H^-GppNHp crystals. (*D*) A surface representation of the binding of Ch-1 (*Left*), Ch-2 (*Middle*), and Ch-3 (*Right*) in KRAS pocket I, close to the switch regions I (red) and II (blue). Full electron density (2Fo-Fc) was found for the three compounds, all depicted as a green mesh. (*E*) An expanded view of the interaction of the compounds with KRAS with the following residues in contact: K5, L6, V7, S39, R41, R41, D54, I55, L56, Y71, and T74.

Our data show that PPIN-1 and PPIN-2 bind to KRAS at the same pocket I as several previously identified compounds (23), but they do not disrupt RAS function. To understand the lack of RAS inhibition of the PPIN compounds, we selected one of our RAS-binding intracellular antibody-derived compounds (Abd-7) able to interfere with RAS PPI in cells (28). We used the computational chemistry suite FORGE (https://www.cresset-group.com/forge/; ref. 34) that employs a ligand comparison method to align and score molecules independently using their shape and electrostatic properties, aiding the understanding of structure–activity relationships. FORGE was used to compare the structures of PPIN-1 and -2 with Abd-7 by performing an alignment based on the surfaces of the compounds only and detected similarities in the lower half of the molecules (Fig. 3 *A* and *B*). The aromatic ring of the benzodioxane moiety in Abd-7 aligned with the terminal biphenyl aryl ring in both PPIN compounds, and the pyridine ring of Abd-7 aligned with the middle aromatic ring of both PPIN compounds. This suggested that the PPIN biphenyl system might act as the anchor to the RAS protein. The alignment with the functional groups on the upper half of the molecules was poor. We used these analyses to design of cross-over compounds linking the common diphenyl-anchoring moiety of PPIN-1 and PPIN-2 to the Abd-7 aniline fragment.

Three cross-over compounds (Fig. 3*C*: Ch-1, Ch-2, and Ch-3) were synthesized and their binding geometries were determined by X-ray crystallography using KRAS_169_^Q61H^ crystal soaking (Fig. 3 *D* and *E* and *SI Appendix*, Table S4). All three compounds showed very similar binding modes to Abd-7 with van der Waals contacts to K5, L6, V7, S39, Y40, R41, D54, I55, L56, G70, Y71, T74, and G75. Ch-1 was found in four of the six KRAS_169_^Q61H^ chains (A, B, C, and F); Ch-2 was again found in three of the six chains (A, B, and C) and Ch-3 was found in four of the six chains (A, B, C, and D). Thus, by combining the anchor constituent of the PPIN compounds with the aniline fragment of Abd-7, we generated a compound series showing good electron density for the entire molecule when bound to KRAS pocket 1.

### The RAS-Binding Cross-Over Compounds Are PPI Inhibitors in Cells.

**Fig. 4.**
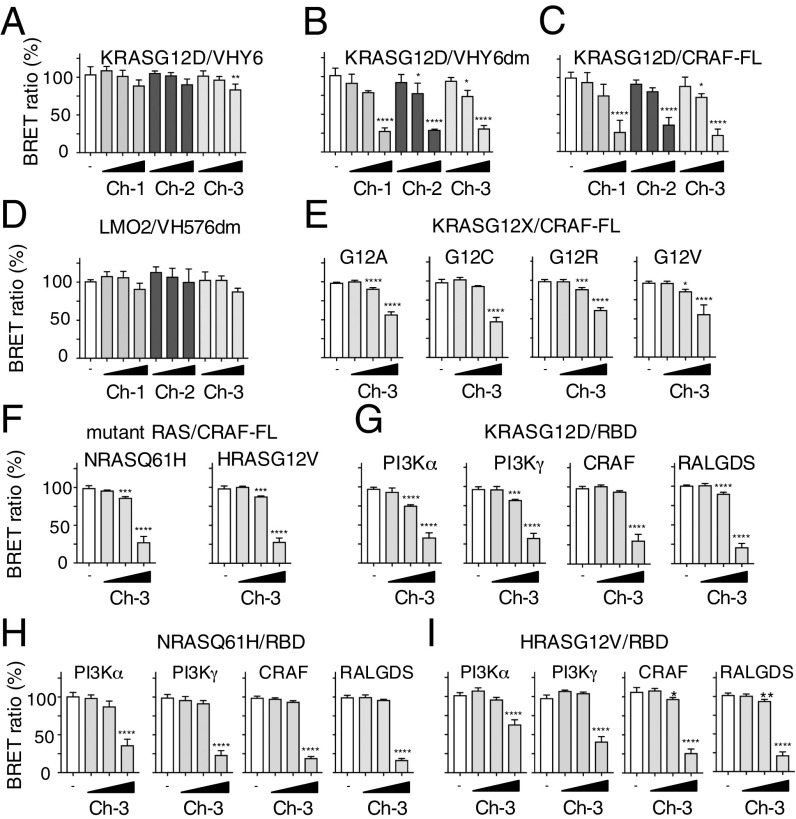
Compound Ch-3 disrupts RAS-effector interactions. Assessment of the inhibition of RAS protein–protein interactions in cells by the chemical series compounds Ch-1, Ch-2, and Ch-3 using different BRET-based RAS biosensor expression vectors. (*A*–*C*) Data from BRET assays using RLuc8-KRAS^G12D^ with either anti-RAS VHY6-GFP^2^ (*A*) with dematured anti-RAS VHY6dm-GFP^2^ (*B*) or with full-length CRAF^FL^-GFP^2^ (*C*). (*D*) Data from BRET assay using a negative control BRET-based biosensor LMO2/VH576dm. The VH576dm is a dematured anti-LMO2 VH. The data are computed relative to cells treated with DMSO vehicle only (open bar) or with Ch-1, Ch-2, or Ch-3 (shaded bars). (*E* and *F*) The effect of the Ch-3 compound on mutant KRAS^G12X^ (*E*) and NRAS^Q61H^ and HRAS^G12V^ (*F*) interactions with CRAF^FL^. (*G*–*I*) The effect of Ch-3 on the interaction of KRAS^G12D^ (*G*), NRAS^Q61H^ (*H*), and HRAS^G12V^ (*I*) with various RAS effector domains (PI3Kα, PI3Kγ, CRAF, and RALGDS). The range of concentration of the compounds was 5, 10, and 20 μM. Each experiment was repeated at least twice (biological replicates). Statistical analyses were performed using a one-way ANOVA followed by Dunnett’s posttests (*P < 0.05, **P < 0.01, ***P < 0.001, ****P < 0.0001). Error bars correspond to mean values ± SD of biological repeats. RLuc8-KRAS, NRAS, and HRAS all comprised full-length RAS components.

The purpose of the compounds was to generate inhibitors of RAS PPI. The ability of the compounds to interfere with RAS-associated PPI was analyzed with our BRET-based RAS biosensor toolbox (31). The interaction of full-length KRAS^G12D^ and either the anti-RAS iDAb VHY6, a dematured version of this iDAb (VHY6dm), or the natural RAS partner CRAF^FL^ was assessed as a dose–response with the three compounds (respectively, Fig. 4 *A–C*). While the interaction of the high-affinity WT iDAb with RAS was minimally affected, even at the highest dose of compound (i.e., 20 μM), both the lower-affinity–dematured iDAb and CRAF binding to RAS were progressively impaired, starting at the lowest dose of 5 μM. No alteration in the BRET signal for the PPI of a nonrelevant protein pair (LMO2-VH576dm) was observed (Fig. 4*D*), confirming that the dose–response effects of the Ch compounds was not due to loss of cell viability.

We also tested the effect of compound Ch-3 in the BRET assay using five different full-length KRAS^G12^ mutations interacting with full-length CRAF (Fig. 4*E*: G12A, G12C, G12R, and G12V) or CRAF RAS-binding domain (RBD) (Fig. 4*G*: G12D). Each of these PPIs was inhibited by the Ch-3 compound in a dose–response assay. Furthermore, the BRET interaction signal between KRAS^G12D^ and either PI3K (α or γ) or RALGDS was inhibited by Ch-3 (Fig. 4*G*), demonstrating that the BRET data are not restricted to KRAS-CRAF interaction. Finally, we show that Ch-3 interferes with NRAS and HRAS isoforms using the BRET biosensor assay. Interactions between full-length NRAS^Q61H^ or HRAS^G12V^ and full-length CRAF (Fig. 4*F*), between NRAS^Q61H^ and RBD for PI3K (α or γ), CRAF, and RALGDS (Fig. 4*H*), or between HRAS^G12V^ and RBD for PI3K (α or γ), CRAF, and RALGDS (Fig. 4*I*) are inhibited in the BRET assay by Ch-3.

**Fig. 5.**
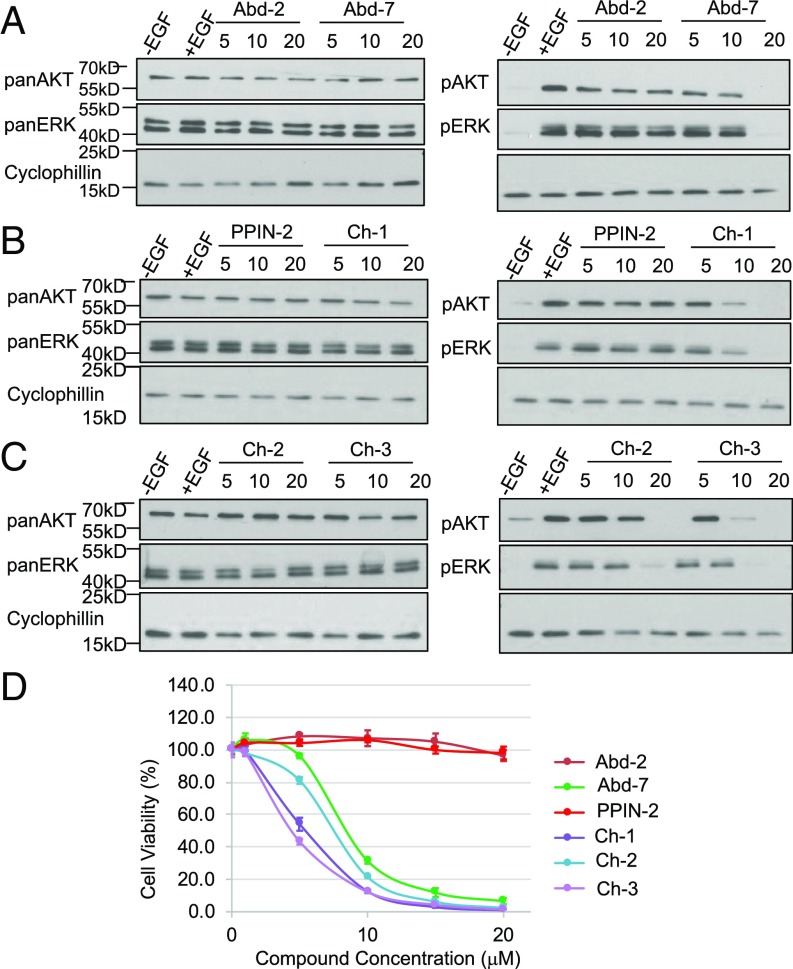
Activity of compounds in a mutant KRAS human cancer cell. The new chemical series compounds Ch-1, -2, and -3 were assessed in two cell-based assays. (*A*–*C*) Western blot analysis of EGF-stimulated DLD-1 cells treated with 5, 10, and 20 μM of Abd-2 or Abd-7 (*A*), PPIN-2 or Ch-1 (*B*), and Ch-2 or Ch-3 (*C*). Cell extracts were fractionated by SDS/PAGE and transferred to PVDF membranes that were incubated with antibodies detecting the indicated proteins. These data are quantitated in *SI Appendix*, Fig. S5. (*D*) DLD-1 cell viability 72 h after treatment with a single application of compound at the indicated concentrations. Viability was determined using the CellTitreGlo method and carried out in triplicate. The data are plotted as normalized cell viability mean with error bars showing SDs.

These BRET data show that the three cross-over compounds can enter cells and reach their target protein (RAS) in the cytoplasmic environment. The BRET assay relies on cotransfected donor and acceptor expression plasmids, and we confirmed this inhibitory capability by testing the effect of the compounds on biomarker phosphorylation in DLD-1 colorectal cancer cells. The cells were incubated with our previously described antibody-derived compounds Abd-2 and Abd-7, the original PPIN-2 compound, or the three cross-over compounds Ch-1, -2, and -3. Phosphorylation of AKT (downstream of RAS-PI3K signaling) or phosphorylation of ERK (downstream of RAS-RAF signaling) was determined following EGF stimulation (Fig. 5 *A–C*). The PPIN-2 (and control Abd-2) had no effect on the levels of phospho-AKT or phospho-ERK even at 20 μM (addressed using Western blotting, Fig. 5 *A–C*), but we found that the three cross-over compounds caused loss of phospho-AKT or phospho-ERK, as did the previously described compound Abd-7. The most potent compound appears to be Ch-3, which invokes an almost complete reduction of phospho-AKT at a concentration of 10 μM while not affecting AKT protein levels (Fig. 5*C*).

The biomarker Western blotting assay was carried out 2.5 h after addition of the compounds, at which time no loss of viability was observed. The survival of DLD-1 cells was determined over 48- and 72-h periods using a dose–response (0–20 μM) (Fig. 5*D*; data shown at 72 h), allowing a calculation of IC_50_ for each compound (*SI Appendix*, Table S5). The previously characterized low-affinity Abd-2 compound does not affect DLD-1 viability over the range of concentrations nor does the PPIN-2 compound. Conversely, the intracellular antibody-derived compound Abd-7 causes loss of viability with IC_50_ of 10.8 μM at 48 h and 8.2 μM at 72 h. The potency of the two of the cross-over compounds (Ch-1 and Ch-3) is improved, relative to Abd-7, as these show IC_50_ at 72 h of 5.3 and 4.5 μM, respectively (*SI Appendix*, Table S5). This increased efficacy of Ch-3 in the challenge of DLD-1 viability matches the most efficacious compound in the signaling biomarker assay.

## Discussion

The *RAS* family of genes is among the most frequently mutated in human cancer (e.g., up to 96% in pancreatic cancer) and therefore an important target for drug development. Targeting the RAS-effector PPI is one possible route to RAS inhibitors. Screening chemical compound libraries per se does not guarantee selection of compounds that will act as PPI inhibitors unless there is a method to guide the screen to functionally specific locations. Strategies are needed that will allow protein-binding compounds to be selected and improved in their properties related to the functional mechanism to be disrupted. In particular, X-ray crystallography of RAS proteins is a key method to determine the location and geometry of bound compounds. We optimized production of KRAS_169_^Q61H^ crystals for soaking of compounds for this purpose. It should be noted that these conditions, in principle, could be used to produce crystals of other RAS mutants suitable for compound-soaking experiments. Surface analysis of KRAS (using the CASTp server: sts.bioe.uic.edu/castp/) confirmed three pockets with internal volumes greater than 80 Å^3^ (*SI Appendix*, Fig. S1*A*: pockets I, III, and IV) and a less deep, more like a shallow groove, pocket (*SI Appendix*, Fig. S1*A*: pocket II). Pocket I was previously identified in silico (29) and was also the site where DCAI was first selected (23). In addition, this pocket is close to the switch region and could act as a point of inhibition for PPI. Furthermore, we have focused on the identification and optimization of compounds inhibiting RAS-effector interactions and not other RAS modulation effects, such as DCAI has shown. Structural comparison of the six chains in the asymmetric unit show that compound binding has a minimal effect on the conformation of the switch regions in RAS protein. Any observed differences are no more than twice the coordinate error (35) and therefore cannot be considered significant. Comparison of the temperature factors between RAS molecules with compound bound and 3GFT (where no compound is present) show no evidence that compound binding results in significant reduction in flexibility of switch II relative to the rest of the molecule, suggesting that any stabilization of the switch regions by compound binding is, at most, slight. Therefore, we conclude that any inhibitory effect observed is due to a disruption of RAS-effector PPI rather than any other modulation of the RAS protein.

We have screened a chemical library that yielded two RAS-binding compounds (the PPINs) and have shown, by crystallography, that they bind to pocket I near to the effector binding sites. However, when these were tested in an orthogonal NMR waterLOGSY binding assay, their binding to KRAS_166_^G12V^ was not impaired by the presence of the anti-RAS intracellular antibody fragment, nor did they interfere with PPI using a cell-based BRET assay (*SI Appendix*, Fig. S4). Crystallography and medicinal chemistry was undertaken to improve the initial PPIN hits using structure-based design combining the crystal information and molecular fragments from two different chemical series, namely PPIN and the Abd series (28). The resulting cross-over compounds illustrate that this strategy guided the conversion of the PPIN RAS-binding compounds to RAS PPI inhibitors in a series (compounds designated Ch-1–3). These compounds have a low molecular weight suitable for further medicinal chemistry to improve drug-like properties and with better ligand efficiencies than their progenitors (*SI Appendix*, Table S5). Furthermore, the compounds have also shown better cell viability results than their progenitors. Their low molecular weight makes them a better starting point for the development of RAS inhibitors based on this promising chemical series.

We have previously shown that the Abd chemical series affected RAF, RAL, and PI3K interactions with RAS (28) and, due to the similarities in binding mode and orientation of the Ch series with the Abd series, we expect the Ch compounds to have a similar range of profiles with other KRAS mutants and also with NRAS and HRAS isoforms. This was confirmed using various KRAS^G12^ mutants and the NRAS^Q61H^ and HRAS^G12V^ mutants with four effector molecules in BRET assays.

This approach shows that compounds binding in pocket I are not necessarily able to inhibit RAS PPI, but synthetically linking components of two classes of RAS-binding compounds can generate new active molecules that inhibit PPI. It should also be noted that the presence of pocket I in nonmutated forms of RAS as well as in mutant RAS (*SI Appendix*, Fig. S1 *B*–*G*) presents a technical challenge for the development of anti-RAS drugs since these will bind to the pocket in WT-activated RAS as well as in mutant RAS. Development of methodologies for specific drug delivery could avoid drug interference with nonmutated RAS. Approaches such as Antibody-Drug Conjugates (ADC) offer one route to avoid drugs entering normal cells (reviewed in ref. 36) by targeting antigens expressed on tumors. While this, in turn, has difficulties, since few surface antigens are tumor-specific, surfaceome studies of tumors (37, 38) can find possible markers or pairs of markers that may be useful for mono- or bispecific ADCs. An alternative approach, based on structure–activity relationships, could be the development of compounds anchoring at pocket I and moving toward the nucleotide-binding region of RAS or perhaps linking the “unselective” but potent compounds identified in pocket I to those binding in pocket II.

In conclusion, our approach demonstrates the importance in drug development of combining assays for PPI with the identification of compounds that bind at important locations but not necessarily with PPI properties. Thus, compounds binding with good potency to the target but not showing any effect on PPI could be utilized in combination chemistry to create new chemical series. A combination of high-resolution crystallography from different chemical series with biophysical competition assays, such as using high-affinity antibody fragments, is thus a powerful way to identify hit compounds of interest in analogous settings. It is also useful in the development of new chemical series, even when initial compounds are inactive, and should allow directed medicinal chemistry for drug development.

## Methods

Detailed methods on protein expression and purification, SPR screening, NMR analysis, crystallography experiments, cell-based assays, and chemical experiments can be found in *SI Appendix*, *Methods*. The atomic coordinates have been deposited in the Protein Data Bank (39–47).

## Supplementary Material

Supplementary File

## References

[1] Cox AD, Fesik SW, Kimmelman AC, Luo J, Der CJ (2014). Drugging the undruggable RAS: Mission possible?. Nat Rev Drug Discov.

[2] Prior IA, Lewis PD, Mattos C (2012). A comprehensive survey of Ras mutations in cancer. Cancer Res.

[3] Simanshu DK, Nissley DV, McCormick F (2017). RAS proteins and their regulators in human disease. Cell.

[4] Gutierrez L, Magee AI, Marshall CJ, Hancock JF (1989). Post-translational processing of p21ras is two-step and involves carboxyl-methylation and carboxy-terminal proteolysis. EMBO J.

[5] Wittinghofer A, Nassar N (1996). How Ras-related proteins talk to their effectors. Trends Biochem Sci.

[6] Bos JL, Rehmann H, Wittinghofer A (2007). GEFs and GAPs: Critical elements in the control of small G proteins. Cell.

[7] Cherfils J, Zeghouf M (2013). Regulation of small GTPases by GEFs, GAPs, and GDIs. Physiol Rev.

[8] Hunter JC (2015). Biochemical and structural analysis of common cancer-associated KRAS mutations. Mol Cancer Res.

[9] Wood KW, Sarnecki C, Roberts TM, Blenis J (1992). Ras mediates nerve growth factor receptor modulation of three signal-transducing protein kinases: MAP kinase, Raf-1, and RSK. Cell.

[10] Scott DE, Bayly AR, Abell C, Skidmore J (2016). Small molecules, big targets: Drug discovery faces the protein-protein interaction challenge. Nat Rev Drug Discov.

[11] Spiegel J, Cromm PM, Zimmermann G, Grossmann TN, Waldmann H (2014). Small-molecule modulation of Ras signaling. Nat Chem Biol.

[12] Whyte DB (1997). K- and N-Ras are geranylgeranylated in cells treated with farnesyl protein transferase inhibitors. J Biol Chem.

[13] Tanaka T, Rabbitts TH (2008). Functional intracellular antibody fragments do not require invariant intra-domain disulfide bonds. J Mol Biol.

[14] Tanaka T, Rabbitts TH (2012). Intracellular antibody capture (IAC) methods for single domain antibodies. Methods Mol Biol.

[15] Cochet O (1998). Intracellular expression of an antibody fragment-neutralizing p21 ras promotes tumor regression. Cancer Res.

[16] Dewhirst MW, Secomb TW (2017). Transport of drugs from blood vessels to tumour tissue. Nat Rev Cancer.

[17] Ostrem JM, Shokat KM (2016). Direct small-molecule inhibitors of KRAS: From structural insights to mechanism-based design. Nat Rev Drug Discov.

[18] Ledford H (2015). Cancer: The Ras renaissance. Nature.

[19] Burns MC (2014). Approach for targeting Ras with small molecules that activate SOS-mediated nucleotide exchange. Proc Natl Acad Sci USA.

[20] Lito P, Solomon M, Li LS, Hansen R, Rosen N (2016). Allele-specific inhibitors inactivate mutant KRAS G12C by a trapping mechanism. Science.

[21] Maurer T (2012). Small-molecule ligands bind to a distinct pocket in Ras and inhibit SOS-mediated nucleotide exchange activity. Proc Natl Acad Sci USA.

[22] Ostrem JM, Peters U, Sos ML, Wells JA, Shokat KM (2013). K-Ras(G12C) inhibitors allosterically control GTP affinity and effector interactions. Nature.

[23] Sun Q (2012). Discovery of small molecules that bind to K-Ras and inhibit Sos-mediated activation. Angew Chem Int Ed Engl.

[24] Winter JJ (2015). Small molecule binding sites on the Ras:SOS complex can be exploited for inhibition of Ras activation. J Med Chem.

[25] Athuluri-Divakar SK (2016). A small molecule RAS-mimetic disrupts RAS association with effector proteins to block signaling. Cell.

[26] Patricelli MP (2016). Selective inhibition of oncogenic KRAS output with small molecules targeting the inactive state. Cancer Discov.

[27] Welsch ME (2017). Multivalent small-molecule Pan-RAS inhibitors. Cell.

[28] Quevedo CE (2018). Small molecule inhibitors of RAS-effector protein interactions derived using an intracellular antibody fragment. Nat Commun.

[29] Grant BJ (2011). Novel allosteric sites on Ras for lead generation. PLoS One.

[30] Sewell H (2014). Conformational flexibility of the oncogenic protein LMO2 primes the formation of the multi-protein transcription complex. Sci Rep.

[31] Bery N (2018). BRET-based RAS biosensors that show a novel small molecule is an inhibitor of RAS-effector protein-protein interactions. eLife.

[32] Johnstone S, Albert JS (2017). Pharmacological property optimization for allosteric ligands: A medicinal chemistry perspective. Bioorg Med Chem Lett.

[33] Lawson ADG, MacCoss M, Heer JP (2018). Importance of rigidity in designing small molecule drugs to tackle protein-protein interactions (PPIs) through stabilization of desired conformers. J Med Chem.

[34] Andersson V (2016). Macrocyclic prodrugs of a selective nonpeptidic direct thrombin inhibitor display high permeability, efficient bioconversion but low bioavailability. J Med Chem.

[35] Cruickshank DWJ (1999). Remarks about protein structure precision. Acta Crystallogr D Biol Crystallogr.

[36] Beck A, Goetsch L, Dumontet C, Corvaïa N (2017). Strategies and challenges for the next generation of antibody-drug conjugates. Nat Rev Drug Discov.

[37] Town J (2016). Exploring the surfaceome of Ewing sarcoma identifies a new and unique therapeutic target. Proc Natl Acad Sci USA.

[38] Martinko AJ (2018). Targeting RAS-driven human cancer cells with antibodies to upregulated and essential cell-surface proteins. eLife.

[39] Cruz-Migoni A (2018). https://www.rcsb.org/structure/6GOD.

[40] Cruz-Migoni A (2018). https://www.rcsb.org/structure/6GOE.

[41] Cruz-Migoni A (2018). https://www.rcsb.org/structure/6GOF.

[42] Cruz-Migoni A (2018). https://www.rcsb.org/structure/6GOG.

[43] Cruz-Migoni A (2018). https://www.rcsb.org/structure/6GOM.

[44] Cruz-Migoni A (2018). https://www.rcsb.org/structure/6GQT.

[45] Cruz-Migoni A (2018). https://www.rcsb.org/structure/6GQW.

[46] Cruz-Migoni A (2018). https://www.rcsb.org/structure/6GQX.

[47] Cruz-Migoni A (2018). https://www.rcsb.org/structure/6GQY.

