# Supplementary material for: Structure-based development of new RAS-effector inhibitors from a combination of active and inactive RAS-binding compounds
Source: Proc Natl Acad Sci U S A. 2019 Jan 25;116(7):2545–50. doi: 10.1073/pnas.1811360116 (PMC6377466; doi:10.1073/pnas.1811360116)
Supplement: Supplementary File [file pnas.1811360116.sapp.pdf]

PNAS
www.pnas.org

Supplementary Information for

Structure-based development of new RAS-effector inhibitors from

a combination of active and inactive RAS-binding compounds.

Abimael Cruz-Migoni [1,2 ^], Peter Canning [1,3 ^], Camilo E. Quevedo [1 ^], Carole J.R. Bataille[4],

Nicolas Bery [1], Ami Miller [1], Angela Russell [4], Simon E.V. Phillips [2,5], Stephen B. Carr[2,5],

Terence H. Rabbitts [1, *]

* Email: terence.rabbitts@imm.ox.ac.uk

This PDF file includes:

Supplementary text (including compound experimental protocols and data)
Figures S1 to S6
Tables S1 to S5
References for SI citations

www.pnas.org/cgi/doi/10.1073/pnas.1811360116

METHODS

Recombinant protein expression for crystallography

$KRAS_{169}^{Q61H}$, and $KRAS_{188}^{G12D}$ cDNAs were cloned into the pRK-172 vector using NdeI and BamHI restriction sites. Genes were placed in-frame with an N-terminal 6 x his-tag and the TEV protease recognition site. $KRAS_{188}^{WT}$ and $KRAS_{188}^{G12V}$ constructs were generated using QuikChange Site-Directed Mutagenesis kit (Agilent Technologies, USA) using pRK-172-$KRAS_{188}^{G12D}$ vector as a template. Plasmids containing pRK-172-6xHis-TEV-$KRAS_{169}^{Q61H}$, $KRAS_{188}^{G12D}$, $KRAS_{188}^{WT}$ or $KRAS_{188}^{G12V}$ sequences were transformed individually into B834(DE3)pLysS cells, which were grown in 25 ml LB medium with 50 µg/ml Carbenicillin and 34 µg/ml Chloramphenicol for 16 hours, before adding to 1L LB medium containing the same antibiotics. Protein expression was induced when cells reached an OD_{600} of 0.6 by addition of 1-thio-β-D-galactopyranoside (IPTG) to a final concentration of 0.5 mM followed by overnight incubation at 16°C. Bacteria were harvested by centrifugation (5180g, 30 mins, 4 °C) resuspended in 60 ml of 50 mM Tris-HCl, pH 7.5, 500 mM NaCl, 5 mM $MgCl_2$ and 10 mM imidazole also containing one EDTA-free protease inhibitor cocktail table (Roche Diagnostics, Mannheim, Germany). Cells were then lysed by sonication using five 30s pulses on power setting 16 with 1 min pauses on ice between pulses and insoluble debris removed by centrifugation (75,600g, 20 mins, and 4 °C). Supernatant was applied to nickel agarose beads (Invitrogen) by gravity, beads were then washed twice using same lysis buffer containing 50 mM imidazole and bound proteins were eluted in 50 mM Tris-HCl, pH 7.5, 500 mM NaCl, 5 mM $MgCl_2$ and 300 mM imidazole. For $KRAS_{188}^{G12D}$,$KRAS_{188}^{WT}$ and $KRAS_{188}^{G12V}$ HIS-tagged TEV protease (1.4mg/ml) was added at a ratio of 1:100 to the eluate and the sample dialysed against 50 mM Tris-HCl, pH 7.5, 500 mM NaCl, 5 mM $MgCl_2$, overnight at 4°C. His-tagged TEV protease and cleaved HIS-tag were removed by reapplication of the digestion to nickel agarose beads (Invitrogen). Samples containing RAS protein were concentrated using Vivapore 10/20 mL concentrator (7.5 kDa molecular weight cut-off; Sartorius Vivapore). The proteins were further purified by gel filtration using a Superdex 75 Increase 10/300 GL column (GE Healthcare, Uppsala, Sweden) equilibrated with 20 mM HEPES pH 8.0, 150 mM NaCl , 5 mM $MgCl_2$ and 1 mM DTT at a flow rate of 0.5 ml/min. Fractions corresponding to RAS were pooled and concentrated to 45-75 mg/ml for crystallization trials. Protein concentration was determined from a theoretical extinction coefficient at 280 nm calculated using ProtParam tool (https://web.expasy.org/protparam/). Protein purity was analyzed by SDS-PAGE stained with Instant Blue (Expedeon). The purification procedure of $KRAS_{188}^{G12D}$, $KRAS_{188}^{WT}$ or $KRAS_{188}^{G12V}$ proteins were the same as for the $KRAS_{166}^{G12V}$ and $KRAS_{169}^{Q61H}$ described above, except gel filtration used the following buffer (25 mM TrisCl, pH 8.0, 100 mM NaCl, 5 mM $MgCl_2$ and 1 mM TCEP). Nucleotide exchange for crystallographic samples was carried out following published procedures (1).

Protein crystallization and structure determination

$KRAS_{169}^{Q61H}$ crystals were grown at 4 °C by mixing 1.5 ul of protein (75 mg/ml) with an equal volume of crystallization buffer before equilibrating against 0.5 ml of crystallization buffer using sitting drop vapor diffusion. Crystal of this variant appeared in drops containing 8-15% w/v Polyethylene Glycol 3350 and 0.2 M lithium citrate pH 5.5. For $KRAS_{188}^{G12D}$ crystals, drops were prepared by mixing 1.5 µl of protein at 45 mg/ml with 1.5 µl of reservoir consisting of 0.1 M TrisCl pH 8.0, 0.2 M NaOAc and 30-35 % PEG 4000 in 24-well Cryschem sitting-drop plates. Crystals of $KRAS_{188}^{WT}$ and $KRAS_{188}^{G12V}$ mutants were obtained in a sitting-drop setup experiment using micro-seeding. $KRAS_{188}^{WT}$ protein and precipitant were mixed in a 1:1 ratio in an initial volume of 2 ul and 0.5 ul of $KRAS_{188}^{G12D}$ micro-crystals were added. $KRAS_{188}^{G12D}$ seed stocks were prepared by mechanical homogenization of crystals using the Seed Bead Kit (Hampton Research) as

described by Luft & DeTitta (2). Micro-crystals were suspended in 50µl reservoir solution containing the seed bead followed by mixing for 3min using a laboratory vortex. The seed-stock solutions were stored at −20°C The same procedure was followed for the generation of $KRAS_{188}^{G12V}$crystals. $KRAS_{188}^{G12V}$ and $KRAS_{166}^{WT}$ crystals were obtained in a crystallization solution of 0.1 M Tris pH 8.0, M NaOAc, 31.4% PEG 4000.
Prior to data collection, crystals were cryo-protected by addition of 20% glycerol to the crystallization buffer, then flash-cooled in liquid nitrogen. For crystal soaking experiments, compounds were added individually (25-50mM of compound in 100%DMSO, with a final DMSO concentration of 6-12% v/v) to the crystallization buffer. Crystals were then transferred to solution containing compound for a minimum of 5 mins. Soaked crystals were also cryo-protected with 20% glycerol and flash-cooled in liquid nitrogen for data collection. X-ray diffraction data were collected at either Diamond Light Source (DLS, Oxfordshire, UK) or European Synchrotron Radiation Facility (ESRF, Grenoble, France). The structure of $KRAS_{169}^{Q61H}$ GPPNHP and $KRAS_{188}^{G12D}$ GPPNHP were solved by molecular replacement using Protein Data Bank (PDB) codes 3GFT and 4DSU, respectively as a search models, within the program Phaser (3). $KRAS_{188}^{WT}$, $KRAS_{166}^{G12V}$ and $KRAS_{188}^{G12V}$ structures were again solved by molecular replacement using the $KRAS_{188}^{G12D}$ GPPNHP solved in this study as a search model. Structures were refined using REFMAC5 (4) and manually corrected using COOT (5). The refined models were validated using MOLPROBITY (6) and Phenix software packages (7) (8). Figures were created using PyMOL (DeLano, 2002). Data collection and refinement statistics are shown in Supplementary Tables 1-4.

Protein expression and purification for SPR fragment screen

The DNA sequence encoding human $KRAS_{166}^{G12V}$ (Uniprot P01116, isoform 4B, residues 1-166) was cloned into the vector pRK, modified to encode an N-terminal hexahistidine tag, TEV cleavage site and AviTag. The LMO2 and the LMO2-VH fusion cDNA were cloned into the vector pOPINS, modified to encode an N-terminal hexahistidine tag, SUMO tag and AviTag. Proteins were expressed in C41(DE3) or Lemo21 cells (NEB) using 0.5mM isopropyl 1-thio-β-D-galactopyranoside (IPTG) for overnight induction at 15ºC. Cells expressing LMO2 were supplemented with 0.1mM $ZnCl_2$. Harvested cells were re-suspended in binding buffer (50mM HEPES pH 7.5, 500mM NaCl, 5% glycerol, 10mM imidazole, 2mM 2-mercaptoethanol) supplemented with Complete Protease Inhibitors (Roche) and disrupted using a cell disruptor (Constant Systems) at 25kPSI, 5ºC. Proteins were partially purified by nickel affinity chromatography, eluting in 500mM imidazole. $KRAS_{166}^{G12V}$ protein was loaded with GppNHp as previously described (1). The N-terminal AviTags on all proteins were biotinylated by treatment with biotin ligase enzyme (BirA) overnight at 4ºC in the presence of ATP, $MgCl_2$ and D-biotin. The hexahistidine and SUMO tags were also cleaved from the LMO2 proteins using SUMO protease. The proteins were further purified by size-exclusion chromatography using either a HiLoad S200 16/600 column or HiLoad S75 16/600 column equilibrated in gel filtration buffer (10mM HEPES pH 7.5, 150mM NaCl, 1mM 2-mercaptoethanol) or PBS on an Akta Avant FPLC system (GE Healthcare).
GST-tagged $KRAS_{166}^{G12V}$ was expressed and purified as previously described REF.

SPR screening

Biotinylated LMO2, KRAS and LMO2-VH fusion were immobilized on a streptavidin chip (GE Healthcare) and the PPI-Net library compounds were passed over the three proteins at 150µM concentration. SPR experiments were carried out using a Biacore T200 instrument (GE Healthcare) at 10 °C to preserve the protein immobilised on the sensor surface. Biotinylated proteins were immobilised on a streptavidin-coated sensor chip (chip

SA, GE Healthcare). The surface was washed with 1M NaCl, 50mM NaOH before immobilising ~4000 response units (RU) of 1µM GppNHp-loaded $KRAS_{166}^{G12V}$ using the 'aim for' program. Control proteins were immobilized at RU level corresponding to an equal number of moles of protein on the sensor surface as 4000 RU of $KRAS_{166}^{G12V}$. In practice this equated to ~4000 RU of LMO2 as the molecular weight (MW) of LMO2 is very close to $KRAS_{166}^{G12V}$. Flow cell 1 was blocked by injecting 10mM biocytin over the surface for 5 minutes at 10µl/min and used as the reference channel. Immobilisation was carried out in HEPES running buffer (10mM HEPES pH 7.4, 150mM NaCl, 0.005% P20, 5mM MgCl2, 10µM $ZnCl_2$). Compound solutions were prepared by transferring 1.5µl PPI-Net stock compounds at 10mM in 100% DMSO into 96-well plates (Greiner) using a multichannel pipette. 98.5µl running buffer with 3.5% DMSO was added to yield a solution of 150µM compound in running buffer with 5% DMSO. Compound solutions were injected over all 4 flow channels for 30 seconds at 30µl/min and dissociation monitored for 60 seconds. A negative control of running buffer with 5% DMSO was run after every 24 cycles. Between injections the flow system was washed with a solution of 50% DMSO. A solvent correction curve was used to correct for the effects of DMSO. Data were referenced, solvent corrected and processed using the T200 evaluation software. Single-point binding levels were exported to Excel. Data were baseline-corrected using the negative control binding levels as a reference and then binding levels measured against $KRAS_{166}^{G12V}$ plotted against binding levels measured against the control proteins. Hit compounds were tested using X-ray crystallography. PPIN-1 and PPNI-2 gave clear electron density in X-ray crystallography.

Recombinant protein expression for NMR
$KRAS_{166}^{G12V}$ was cloned and isolated the same way as described in the recombinant protein expression for crystallization. $KRAS_{166}^{G12V}$ cDNA was cloned into the pGEX vector in-frame with an N-terminal Glutathione-S transferase (GST) tag. pGEX-GST-$KRAS^{G12V}$ was transformed into *E.coli* BL21 (DE3) cells. Bacterial cells were cultured at 37°C to an OD_{600} of 0.5 and induced with IPTG (isopropyl 1-thio-beta-D-galactopyranoside, final concentration 0.1mM) at 16°C overnight. The bacteria cultures were harvested by centrifugation and the cell pellets re-suspended in 50 mM Tris-HCl pH8.0, 140 mM NaCl, 1 mM mercaptoethanol supplemented with complete protease inhibitor (Roche). The GST-fusion proteins were purified by glutathione-sepharose column chromatography (GE Healthcare) and eluted with 50 mM Tris-HCl pH8.0, 10 mM reduced glutathione, 1 mM mercaptoethanol, 5 mM $MgCl_2$.
$KRAS_{166}^{G12V}$ protein samples were concentrated using Vivapore 10/20 mL concentrator (7.5 kDa molecular weight cut-off; Sartorius Vivapore) to a final volume of approximately 2 mL. RAS proteins were further purified by gel filtration on a HiLoad Superdex 75 10/300GL column (GE Healthcare) in a buffer containing 10 mM PBS pH 7.4, 5 mM $MgCl_2$ at a flow rate of 0.5 mL/min. Protein concentration was determined by extinction coefficient (ε_{280} = 54780 $M^{-1}cm^{-1}$). Protein purity was analysed by SDS-PAGE stained with Coomassie Brilliant Blue. Nucleotide exchange for crystallographic samples was carried out following published procedures (1)

NMR spectroscopy
NMR spectra were recorded on Bruker Avance spectrometers (AVII400 or AVIII400) in the deuterated solvent stated. The field was locked by external referencing to the relevant deuteron resonance. Chemical shifts (δ) are reported in parts per million (ppm) referenced to the solvent peak. The multiplicity of each signal is indicated by: s (singlet); br. s (broad singlet); d (doublet); t (triplet); dd (doublet of doublets); tt (triplet of triplets); or m (multiplet).

The number of protons (n) for a given resonance signal is indicated by nH. Coupling constants (J) are quoted in Hz and are reported to the nearest 0.1 Hz.

WaterLOGSY experiments

WaterLOGSY experiments (9) were conducted at a ^{1}H frequency of 600 MHz using a Bruker Avance spectrometer equipped with a BBI probe. All experiments were conducted at 298 K. 3 mm diameter NMR tubes with a sample volume of 200 µL were used in all experiments. Solutions were buffered using an H_2O PBS buffer corrected to pH 7.4 and 5mM $MgCl_2$. The sample preparation is exemplified as follows, the compound (10 µL of a 10 nM solution in DMSO-*d*$_6$) was added to an eppendorf before sequential addition of the H_2O PBS buffer (163.6 µL), D_2O (20 µL), and His-KRAS$_{166}^{G12V}$-GppNHP loaded protein (6.4 µL, 311.8 µM). The resulting solution was spun to ensure full mixing and transferred to a 3 mm NMR tube before the run. For a competition experiment using Y6-ScFv VH, the preparation was carried out in a similar manner; the compound (10 µL of a 10 nM solution in DMSO-*d*$_6$) was added to an eppendorf before sequential addition of the H_2O PBS buffer (146.4 µL), D_2O (20 µL), protein (6.4 µL, 311.8 µM) and Y6-ScFv (17.2 µL, 116.6 µM). The resulting solution was spun and transferred to a 3 mm NMR tube before the run. Negative controls (compound alone, without the protein) were prepared in a similar manner, in order to obtain an end volume of 200 µL.

FORGE Analysis

Alignments of the selected compounds were performed following the wizard menu in FORGE (http://www.cresset-group.com/forge/ (10)). Mol2 files of the Abd7 file was uploaded as the reference molecule. The protonation state for each molecule was selected by FORGE. Mol2 files for the selected molecules to be aligned were uploaded in the program (PPIN-1 and PPIN2). No other parameters were uploaded in the program until the selection for the results quality window appeared. For simplicity, no field alignments were used, only alignments from the compounds surfaces were selected from the highest score solutions to illustrate the results.

Chemical synthesis and characterization

All solvents and reagents were used as supplied (analytical or HPLC grade) without prior purification. Water was purified by an Elix® UV-10 system. Brine refers to a sat. aq. solution of sodium chloride. *In vacuo* refers to the use of a rotary evaporator attached to a diaphragm pump. Thin layer chromatography was performed on aluminium plates coated with 60 F254 silica. Plates were visualised using UV light (254 nm) or 1% aq. $KMnO_4$. Flash column chromatography was performed on Kieselgel 60M silica in a glass column. NMR spectra were recorded on Bruker Avance spectrometers (AVII400, AVIII 400, AVIIIHD 600 or AVIII 700) in the deuterated solvent stated. The field was locked by external referencing to the relevant deuteron resonance. Chemical shifts (δ) are reported in parts per million (ppm) referenced to the solvent peak. ^{1}H spectra reported to two decimal places, and ^{13}C spectra reported to one decimal place, and coupling constants (J) are quoted in Hz (reported to one decimal place). The multiplicity of each signal is indicated by: s (singlet); br. s (broad singlet); d (doublet); t (triplet); q (quartet); dd (doublet of doublets); td (triplet of doublets); qt (quartet of triplets); or m (multiplet). Low-resolution mass spectra were recorded on an Agilent 6120 spectrometer from solutions of MeOH. Accurate mass measurements were run on either a Bruker MicroTOF internally calibrated with polyalanine, or a Micromass GCT instrument fitted with a Scientific Glass Instruments BPX5 column (15 m x 0.25 mm) using amyl acetate as a lock mass, by the mass spectrometry department of the Chemistry Research Laboratory, University of Oxford, UK.; m/z values are reported in Daltons.

NMR spectra for the compounds are shown in Figure S6.

General procedure A

The requisite halogen (600 mg, 2.79 mmol, 1.0 eq.), K_2CO_3 (1.16 g, 8.37 mmol, 3.0 eq.), the requisite boronic acid (572 mg, 3.07 mmol, 1.1 eq.), and Pd(dppf)Cl_2 (100 mg, 0.140 mmol, 5 mol%) were added sequentially to a microwave vial equipped with a magnetic stirrer bar. The reaction vessel was fitted with a rubber septum and purged with N_2 for 5 min, before addition of a degassed solution of 1,4-dioxane/water (5:1, 8 mL) *via* syringe. The vial was then sealed and the reaction heated to 100 °C for 18 h. The mixture was cooled down, diluted with EtOAc (30 mL), and washed with a 50/50 solution of water and brine (2 x 30 mL). The organic phase was dried (Na_2SO_4) and concentrated *in vacuo.* Purification by column chromatography on silica gel (solvents as stated) afforded the desired product.

General procedure B

The requisite halogen (75 mg, 0.272 mmol, 1.0 eq.), Cs_2CO_3 (266 mg, 0.866 mmol, 3.0 eq.), the requisite amine (53 mg, 0.354 mmol, 1.3 eq.), XPhos (13 mg, 0.027 mmol, 10 mol%) and Pd(OAc)$_2$ (3 mg, 0.014 mmol, 5 mol%) were added sequentially to a microwave vial equipped with a magnetic stirrer bar. The reaction vessel was fitted with a rubber septum and purged with N_2 for 5 min, before addition of a degassed solution of 1,4-dioxane (3 mL) *via* syringe. The vial was then sealed and the reaction heated to 100 °C for 24 h. The mixture was cooled down, diluted with EtOAc (30 mL), and washed with a 50/50 solution of water and brine (2 x 30 mL). The organic phase was dried (Na_2SO_4) and concentrated *in vacuo.* Purification by column chromatography on silica gel (solvents as stated) afforded the desired product.

4'-chloro-3-methoxy-1,1'-biphenyl (1)

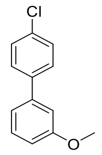

Following **General Procedure A**, 3-bromoanisole (200 mg, 1.07 mmol) and 4-chlorobenzylboronic acid (185 mg, 1.18 mmol) afforded the title product **1** (231 mg, 99%) as a clear oil that solidified on standing after purification on silica gel (EtOAc:pentane (1:99)).

^{1}H NMR (400 MHz, $CDCl_3$) δ = 7.52 (2H, dd, *J* 8.8, 0.7), 7.41 (2H, dd, *J* 8.8, 0.7), 7.36 (1H, dd, *J* 7.6, 0.7), 7.15 (1H, dquin, *J* 7.6, 0.7), 7.09 (1H, dt, *J* 2.6, 1.4), 6.92 (1H, ddt, *J* 8.3, 2.4, 1.0), 3.88 (3H, s); ^{13}C NMR (75 MHz, $CDCl_3$) δ = 160.0, 141.5, 139.5, 133.5, 129.9, 128.9, 128.4, 119.5, 112.9, 112.8, 55.3; LRMS (ESI+) 219.1 $(M+H)^+$.

***N*-(4-((dimethylamino)methyl)phenyl)-3'-methoxy-[1,1'-biphenyl]-4-amine (Ch-1)**

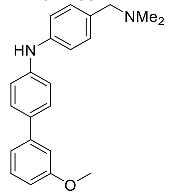

Following **General Procedure B**, **A** (155 mg, 0.711 mmol) and 4-amino-*N*,*N*-dimethylbenzylamine (128 mg, 0.853 mmol) afforded the title product **Ch-1** (209 mg, 88%)

as a yellow oil that solidified on standing after purification on silica gel ($MeOH:CH_2Cl_2$ (1:9)).

^1H NMR (600 MHz, MeOD) δ = 7.50 (2H, d, *J* 8.4), 7.30 (1H, t, *J* 8.0), 7.20 (2H, d, *J* 8.3), 7.16 (2H, d, *J* 8.4), 7.15 (2H, d, *J* 8.8), 7.11 (2H, d, *J* 8.3), 6.83 (1H, dd, *J* 8.3, 2.4), 3.84 (3H, s), 3.51 (2H, s), 2.32 (6H, s), NH was not observed; ^{13}C NMR (125 MHz, MeOD) δ = 161.7, 145.0, 144.7, 134.3, 132.1, 130.9, 129.1, 128.8, 119.8, 118.7, 118.3, 113.1, 113.0, 64.3, 55.8, 44.8; LRMS (ESI+) 333.1 $(M+H)^+$; HRMS (ESI^+) [$C_{22}H_{25}N_2O$] requires 333.4550, found 333.4538;

4-chloro-3,3'-dimethoxy-1,1'-biphenyl (2)

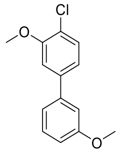

Following **General Procedure A**, 3-bromoanisole (200 mg, 1.07 mmol) and 4-chloro-3-methoxyphenyl boronic acid (220 mg, 1.18 mmol) afforded the title product **2** (232 mg, 99%) as a beige solid after purification on silica gel (EtOAc:pentane (1:99)).

^1H NMR (600 MHz, $CDCl_3$) δ = 7.42 (1H, dd, *J* 7.7, 0.7), 7.37 (1H, t, *J* 7.9), 7.16 (1H, dt, *J* 7.6, 0.9), 7.13 (1H, s), 7.11 (1H,ddd, *J* 11.4, 9.5, 2.0), 7.10 (1H, t, *J* 2.4), 6.93 (1H, dd, *J* 8.3, 2.6), 3.98 (3H, s), 3.88 (3H, s); ^{13}C NMR (125 MHz, $CDCl_3$) δ = 160.0, 155.1, 141.9, 141.2, 130.3, 129.9, 121.8, 120.1, 119.8, 113.1, 112.8, 111.1, 56.2, 55.4; LRMS (ESI+) 249.7 $(M+H)^+$.

***N*-(4-((dimethylamino)methyl)phenyl)-3,3'-dimethoxy-[1,1'-biphenyl]-4-amine (Ch-2)**

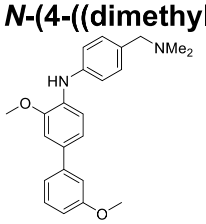

Following **General Procedure B**, **2** (185 mg, 0.746 mmol) and 4-amino-*N*,*N*-dimethylbenzylamine (134 mg, 0.895 mmol) afforded the title product **Ch-2** (226 mg, 91%) as a yellow oil after purification on silica gel ($MeOH:CH_2Cl_2$ (1:9)).

^1H NMR (600 MHz, MeOD) δ = 7.30 (1H, t, *J* 7.9) 7.29 (1H, d, *J* 7.9), 7.20 (2H, d, *J* 8.4), 7.18 (1H, d, *J* 2.0), 7.17 (1H, ddd, *J* 7.7, 1.7, 0.9), 7.14-7.12 (4H, m), 6.84 (1H, ddd, *J* 8.3, 2.6, 0.9), 3.94 (3H, s), 3.83 (3H, s), 3.47 (2H, s), 2.29 (6H, s), NH was not observed; ^{13}C NMR (125 MHz, MeOD) δ = 161.7, 151.0, 144.6, 144.2, 134.9, 133.8, 132.0, 130.9, 129.7, 120.4, 120.1, 118.9, 117.1, 113.4, 113.0, 110.9, 64.4, 58.4, 55.9, 44.9; LRMS (ESI+) 333.1 $(M+H)^+$; HRMS (ESI^+) [$C_{23}H_{27}N_2O_2$] requires 363.4810, found 363.4821.

4-chloro-2-fluoro-3,3'-dimethoxy-1,1'-biphenyl (3)

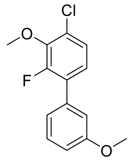

Following **General Procedure A**, 3-bromoanisole (200 mg, 1.07 mmol) and 4-chloro-2-fluoro-3-methoxyphenylboronic acid (241 mg, 1.18 mmol) afforded the title product **3** (272 mg, 96%) as a yellow oil after purification on silica gel (EtOAc:pentane (1:99)).

^1H NMR (600 MHz, $CDCl_3$) δ = 7.38 (1H, t, *J* 7.8), 7.22 (1H, dd, *J* 8.4, 1.8), 7.10 (1H, dd, *J* 8.6, 7.6), 710-7.09 (1H, m), 7.06 (1H, dd, *J* 2.7, 1.5), 6.95 (1H, ddd, *J* 8.3, 2.4, 1.0), 4.01 (3H, d, *J* 1.0), 3.86 (3H, s); ^{19}F NMR (565 MHz, $CDCl_3$) δ = -132.4 (d, *J* 8.0); ^{13}C NMR (125 MHz, $CDCl_3$) δ =159.6, 154.4, 152.7, 144.8 (d, *J* 14.3), 136.0, 129.5, 129.3 9d, *J*

13.2), 127.5 (d, *J* 2.2), 125.1 (d, *J* 3.3), 124.8 (d, *J* 3.3), 121.3 (d, *J* 2.2), 114.6 (d, *J* 2.2), 113.6, 61.6 (d, *J* 4.4), 55.3; LRMS (ESI+) 267.7 $(M+H)^+$.

***N*-(4-((dimethylamino)methyl)phenyl)-2-fluoro-3,3'-dimethoxy-[1,1'-biphenyl]-4-amine (Ch-3)**

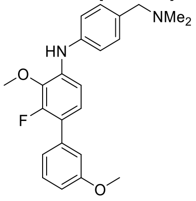

Following **General Procedure B**, **3** (110 mg, 0.414 mmol) and 4-amino-*N*,*N*-dimethylbenzylamine (75 mg, 0.497 mmol) afforded the title product **Ch-3** (226 mg, 91%) as a yellow oil after purification on silica gel (MeOH:CH_2Cl_2 (1:9)).

^{1}H NMR (600 MHz, MeOD) δ = 7.31 (1H, t, *J* 8.0), 7.24 (2H, d, *J* 8.3), 7.17 (2H, d, *J* 8.3), 7.08 (2H, td, *J* 8.6, 1.1), 7.05-7.03 (2H, m), 6.88 (1H, ddd, *J* 8.3, 2.6, 0.8), 3.91 (3H, d, *J* 0.9), 3.82 (3H, s), 3.49 (2H, s), 2.29 (6H, s), NH was not observed; ^{19}F NMR (565 MHz, MeOD) δ = -137.7 (d, *J* 8.2); ^{13}C NMR (125 MHz, MeOD) δ = 161.3, 155.1 (d, *J* 243.2), 143.7, 139.4 9d, *J* 4.4), 138.8, 138.6 (d, *J* 14.3), 132.0, 131.0, 130.5, 125.6 (d, *J* 4.4), 122.6 (d, 12.1), 122.3 (d, *J* 3.3), 120.1, 115.6 (d, *J* 3.3), 113.6, 112.3 (d, *J* 2.2), 64.4, 61.8 (d, *J* 4.4), 55.9, 45.0; LRMS (ESI+) 381.3 $(M+H)^+$; HRMS (ESI^+) [$C_{23}H_{26}FN_2O_2$] requires 381.4714, found 381.4702.

Tissue culture

HEK293T and DLD-1 cells and were grown in DMEM, high glucose, GlutaMax™ medium (Life Technologies) supplemented with 10% FBS (Sigma) and 1% Penicillin/Streptomycin (Life Technologies). Cells were grown at 37°C with 5% CO_2. The KRAS genotype in DLD-1 cells (heterozygous for $KRAS^{G13D}$/KRAS wild type) was confirmed by RNA analysis and sequencing (11).

BRET bioassay

The BRET-based RAS biosensors are built by fusing a donor molecule, a variant of the *Renilla* Luciferase (RLuc8), to full-length mutant RAS ($KRAS^{G12A}$, $KRAS^{G12C}$, $KRAS^{G12D}$, $KRAS^{G12V}$, $KRAS^{G12R}$, $NRAS^{Q61H}$ and $HRAS^{G12V}$ mutants) and an acceptor molecule, GFP^2, to RAS binders (iDAb RAS) or RAS effectors (full-length CRAF; PI3Kα, PI3Kγ, CRAF RBDs and RALGDS RA) (11). A BRET signal (or BRET ratio) occurs only when the donor (e.g. RLuc8-$KRAS^{G12D}$) and acceptor (e.g. GFP^2-$CRAF^{FL}$) molecules are in close proximity (≤ 10 nm). A competition assay involves the addition of a competitor (i.e. anti-RAS compounds) and is used to determine whether an inhibitor can interfere with RAS protein-protein interactions (PPIs). If the compounds impede RAS PPIs, a BRET signal decreased is monitored; otherwise the BRET signal is unchanged.

The BRET assay was performed as described in detail elsewhere (11). Briefly, 650,000 HEK293T cells were transfected with donor plasmid (RLuc8-mutant RAS) and acceptor plasmids (fusions of RAS binders/effectors with GFP^2) to allow the assessment of inhibitors of RAS-effector interaction. The transfection was carried out using Lipofectamine 2000 transfection reagent (Thermo-Fisher). Cells were detached 24 hours later and washed with PBS and seeded in a white 96 well plate (clear bottom, PerkinElmer, cat#6005181) in OptiMEM no phenol red medium (Life Technologies) supplemented with 4% FBS. Cells were left for 4 hours at 37 °C before adding compounds. 10 mM stock compounds in 100% DMSO were diluted in OptiMEM no red phenol + 4% FBS to reach 10X the final concentration (2% DMSO for each concentration). The final concentrations in the cells were 0, 5, 10 and 20 μM (with final 0.2% DMSO each). Quadruplicates were

performed for each point. Cells were left for an additional 20 hours at 37 °C before the BRET2 signal reading directly after addition of Coelenterazine 400a substrate (10 μM final) to cells (Cayman Chemicals, cat#16157). BRET2 reading was done on an Envision instrument (2103 Multilabel Reader, PerkinElmer) with the BRET2 Dual Emission optical module (515 nm ± 30 and 410 nm ± 80; PerkinElmer) or with a CLARIOstar instrument. The BRET ratio corresponds to the light emitted by the GFP^2 acceptor constructs (515 nm ± 30) upon addition of Coelenterazine 400a divided by the light emitted by the RLuc8 donor constructs (410 nm ± 80) with subtraction of the background (same ratio but with RLuc8 construct alone transfected in cells). The normalized BRET ratio is the BRET ratio normalized to the DMSO negative control and calculated as follows: ($BRET_{compound}$ / $BRET_{DMSO}$) x 100, where $BRET_{compound}$ correspond to the BRET ratio for the compound-treated cells, $BRET_{DMSO}$ to the DMSO-treated cells. Each experiment was repeated at least twice.

Quantification and statistical analysis
Statistical analyses of the BRET assays were performed using a one-way ANOVA followed by Dunnett's post-tests (*P<0.05, **P<0.01, ***P<0.001, ****P<0.0001) using GraphPad Prism 7 software.

Biomarker Western blot assay
DLD-1 cells were seeded at 4.5 x 10^5 cells per well in 6-well plates and incubated overnight. The media was aspirated and the cells were washed twice with PBS, before 2ml of serum free DMEM was added to the cells, which were incubated for a further 24 hours. The following day the media was replaced with compound containing media (0-20uM compound, with 0.2% DMSO) and the cells were incubated under culture conditions for 2.5 hours. The cells were stimulated with 50 ng/ml EGF for 10 min at 37°C. The cells were washed with cold PBS and were lysed using RIPA buffer (50mM Tris, pH 8.0, 150 mM NaCl, 1% Triton X-100, 0.5% sodium deoxycholate, 0.1% SDS) containing 1 mM DTT, Complete™ EDTA-free protease inhibitor and PhosSTOP™ (Roche). Cell lysates were sonicated using a Bioruptor® Pico Sonication System (Diagenode) and the protein concentration of each sample was quantified using the Pierce BCA Protein Assay Kit (Thermo Fisher Scientific). 10ug of protein was electrophoresed on a 12% Bis-Tris gel and bands were subsequently transferred to a Amersham™ Hybond® P 0.45 μm PVDF membranes (GE Healthcare). The membranes were blocked with 10%-BSA-TBST or 5%-milk-TBST before being incubated overnight at 4°C with appropriate antibodies (anti-phospho-p44/22 MAPK (ERK1/2) (1/5000, CST, 9101S), anti-p44/42 MAPK (total ERK1/2) (1/1000, CST, 9102S), anti-phospho-AKT S473 (1/2000, CST, 4058S), anti-AKT (1/2000, CST, 9272S), anti-cyclophilin B (1/1000, Abcam, 178397)). The membranes were washed with TBST and incubated with anti-rabbit IgG HRP-linked (1/2500, CST, 7074S) secondary antibody for 2 hours at room temperature. Following thorough washing the membranes were developed using Pierce™ ECL Western Blotting Substrate (Thermo Fisher Scientific) and CL-XPosure™ films (Thermo Fisher Scientific).

Cell viability assays
DLD-1 cells were seeded in culture media at 10,000 cells per well in ViewPlates-96 microplates (PerkinElmer) and allowed to adhere overnight at 37 °C, 5% CO_2. 10mM stock compounds, solubilised in DMSO, were diluted in culture media at final concentrations ranging from 0-20μM, each containing 0.2% DMSO. The media on the cells was removed and 100ul of the compound containing media was added. Quadruplicates were set up for each compound concentration. The cells were incubated under standard culture

conditions for a further 72 hours. Cellular viability was assessed using the CellTiterGlo Luminescent Cell Viability Assay (Promega) according to the manufacturers' instructions. CellTiterGlo was added (50% v/v) to the cells, and the plates incubated for 10 minutes prior to luminescent detection using an Envision 2103 Multilable Microplate Reader (PerkinElmer). The luminescence signals obtained from the compound treated cells were normalized against the signal obtained for the DMSO-only treated cells. The IC_{50} values were generated by non-linear regression using GraphPad Prism 7 software (GraphPad Inc).

PDB files

$KRAS_{188}^{WT}$ GppNHp	**PDB ID: 6GOD**
$KRAS_{188}^{G12V}$ GppNHp	**PDB ID: 6GOE**
$KRAS_{188}^{G12D}$ GppNHp	**PDB ID: 6GOF**
$KRAS_{169}^{Q61H}$ GppNHp	**PDB ID: 6GOG**
$KRAS_{169}^{Q61H}$ GppNHp-PPIN-1	**PDB ID: 6GOM**
$KRAS_{169}^{Q61H}$ GppNHp-PPIN-2	**PDB ID: 6GQT**
$KRAS_{169}^{Q61H}$ GppNHp-Ch-1	**PDB ID: 6GQW**
$KRAS_{169}^{Q61H}$ GppNHp-Ch-2	**PDB ID: 6GQX**
$KRAS_{169}^{Q61H}$ GppNHp-Ch-3	**PDB ID: 6GQY**

Suplementary Figures

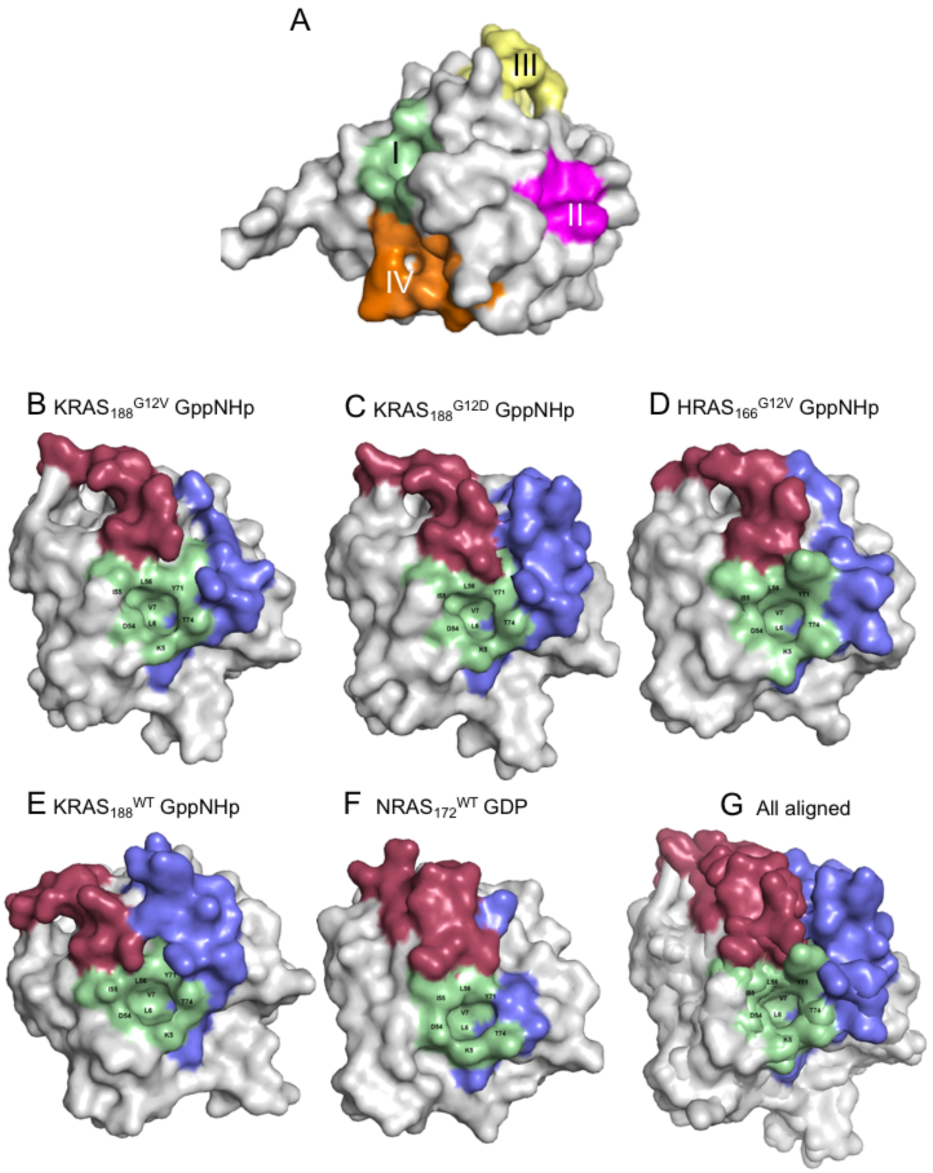

Figure S1. Comparison of potential compound-binding pockets in RAS proteins (wild type and mutant forms)

The structural data for the different RAS proteins and different length proteins has been analyzed to assess potential binding pockets. Four possible pockets exist in full length RAS proteins (Panel A). Pocket I is a hydrophobic pocket near the switch regions; pocket II is a shallow pocket; pocket III is the nucleotide binding pocket and pocket IV is found at the carboxy terminus (evident when full length Ras proteins were analyzed. Pocket I (Sun et al, 2012) is found in K, H and NRAS and different mutant and wild type proteins (Panels B-F) and the topology is very similar in all the proteins as shown by the multi-overlay in Panel G. The panels are B. full length KRAS$_{188}^{G12V}$ (this paper), C. KRAS$_{188}^{G12D}$ (Maurer et al., 2012) and this paper, D. HRAS$_{166}^{G12V}$ (2VH5 KRAS$_{166}^{G12V}$-scFv HRAS) (Tanaka et al., 2007), E. wild type (WT) KRAS$_{188}$ (this paper) and F. full length, wild type NRAS$_{172}$ PDB 3CON. Panel G is a super-imposition of the five structures. Switch I is shown in maroon, switch II in blue and pocket I in green.

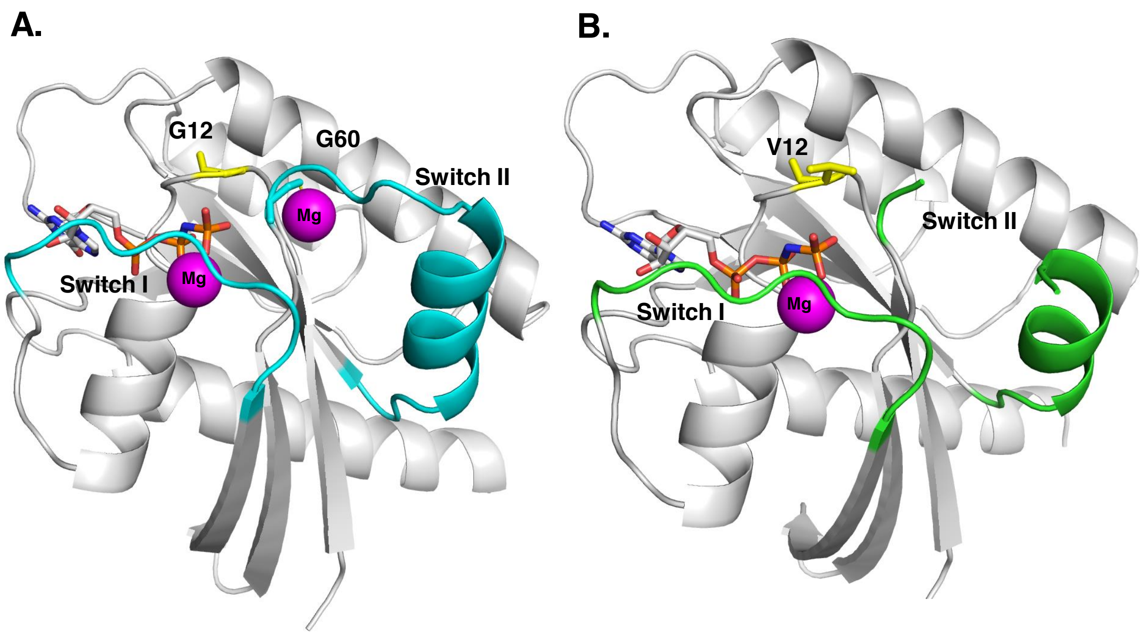

Figure S2. Structure of wild type $KRAS_{188}$ and $KRAS_{188}^{G12V}$ isoforms 4B

Panel A. Ribbon representation of wild type $KRAS_{188}$-GppNHp with the switch regions shown in cyan illustrating the interaction of G60 (depicted in cyan) with the second magnesium ion and stabilizing switch II. Panel B. Ribbon representation of $KRAS_{188}^{G12V}$-GppNHp (switch regions in green). Residues 62 to 66 of switch II are not shown as they are disordered in the maps.

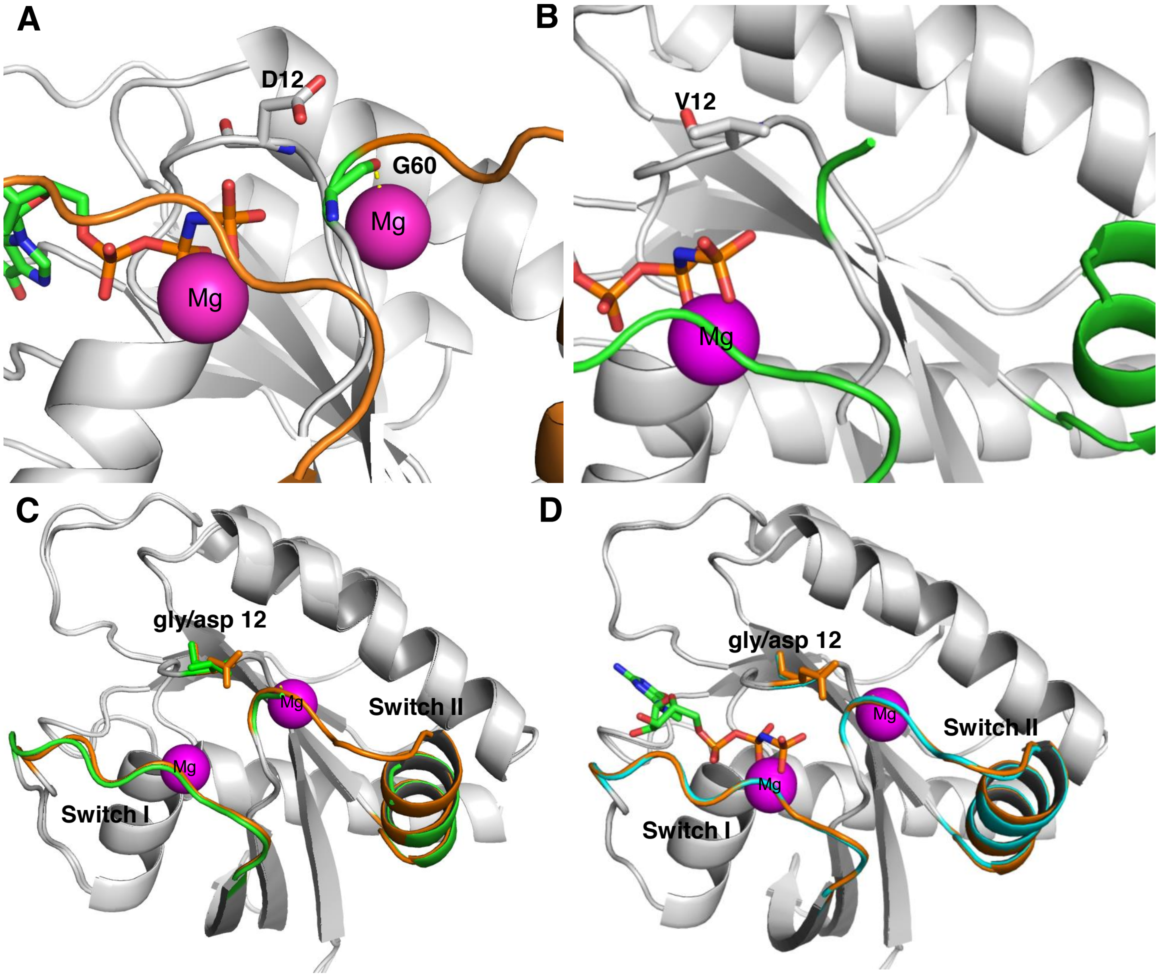

Figure S3. Comparison of the structures of the mutant forms of $KRAS_{188}^{G12D}$ and $KRAS_{188}^{G12V}$

The structures of $KRAS_{188}^{G12D}$ and $KRAS_{188}^{G12V}$ (isoform 4B) are compared in the switch regions with bound GTP-analogue GppNHp.

PANEL A. $KRAS_{188}^{G12D}$ ribbon representation, with Switch I and II in orange, illustrating the interactions of a second Mg ion with G60 found in switch II which stabilize the switch II region. Mg are shown as large magenta spheres.

PANEL B. $KRAS_{188}^{G12V}$ ribbon representation for the switch II region (in green). No second Mg ion was identified in these crystals consistent with an increase in the flexibility of the switch II region of the protein.

PANEL C. Ribbon diagram of an alignment of the $KRAS_{188}^{G12D}$ and G12V. The difference between these structures is the flexibility of the switch II region. $KRAS_{188}^{G12D}$ (switch II shown in orange) has a stable switch II due to the presence of an extra Mg, $KRAS_{188}^{G12V}$ (switch shown in green) has an unstable switch II because of the lack of a second Mg ion around that region.

PANEL D. Ribbon diagram of an alignment of the wild type $KRAS_{188}$ and $KRAS_{188}^{G12D}$. There are no clear differences between these structures, both contain two Mg atoms that gives a higher degree of stability to the switch II regions and the folding is almost identical.

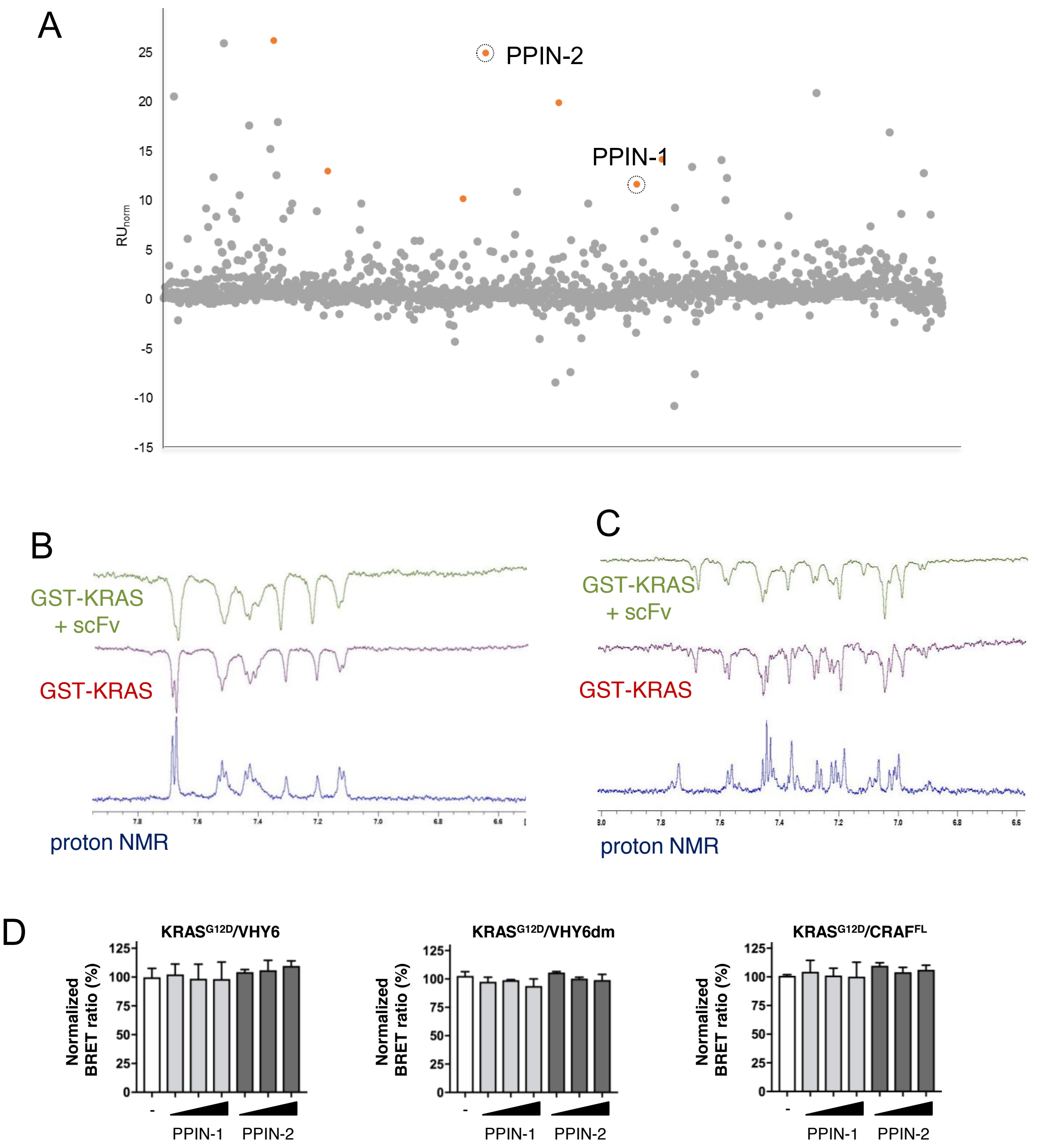

Figure S4. Isolation and characterization of PPIN-1 and -2

The PPI-net compound library (from Prof. Andy Wilson) was screened by SPR at a single concentration and hits PPIN-1 and PPIN-2 identified (panel A, x axis is arbitrary compound position) by binding to KRAS^{G12V}-GppNHp) but not to LMO2 proteins. PPIN-1 and 2 were subjected to waterLOGSY analysis (respectively panel B and C) with GST-KRAS (middle, red trace) or GST-KRAS + anti-RAS scFv (top, green trace). The lower trace (blue) is the proton NMR of the compound alone. Panel D show data from BRET assays using RLuc8-KRAS^{G12D} with either anti-RAS VHY6-GFP^2 (left), with de-matured anti-RAS VHY6-GFP^2 (middle) or with GFP^2-CRAF^{FL} (right). The data are computed relative to cells treated with DMSO vehicle only (open boxes) or PPIN-1 (light shaded boxes) and PPIN-2 (dark shaded boxes). The range of concentration of the compounds was 5, 10 and 20μM.

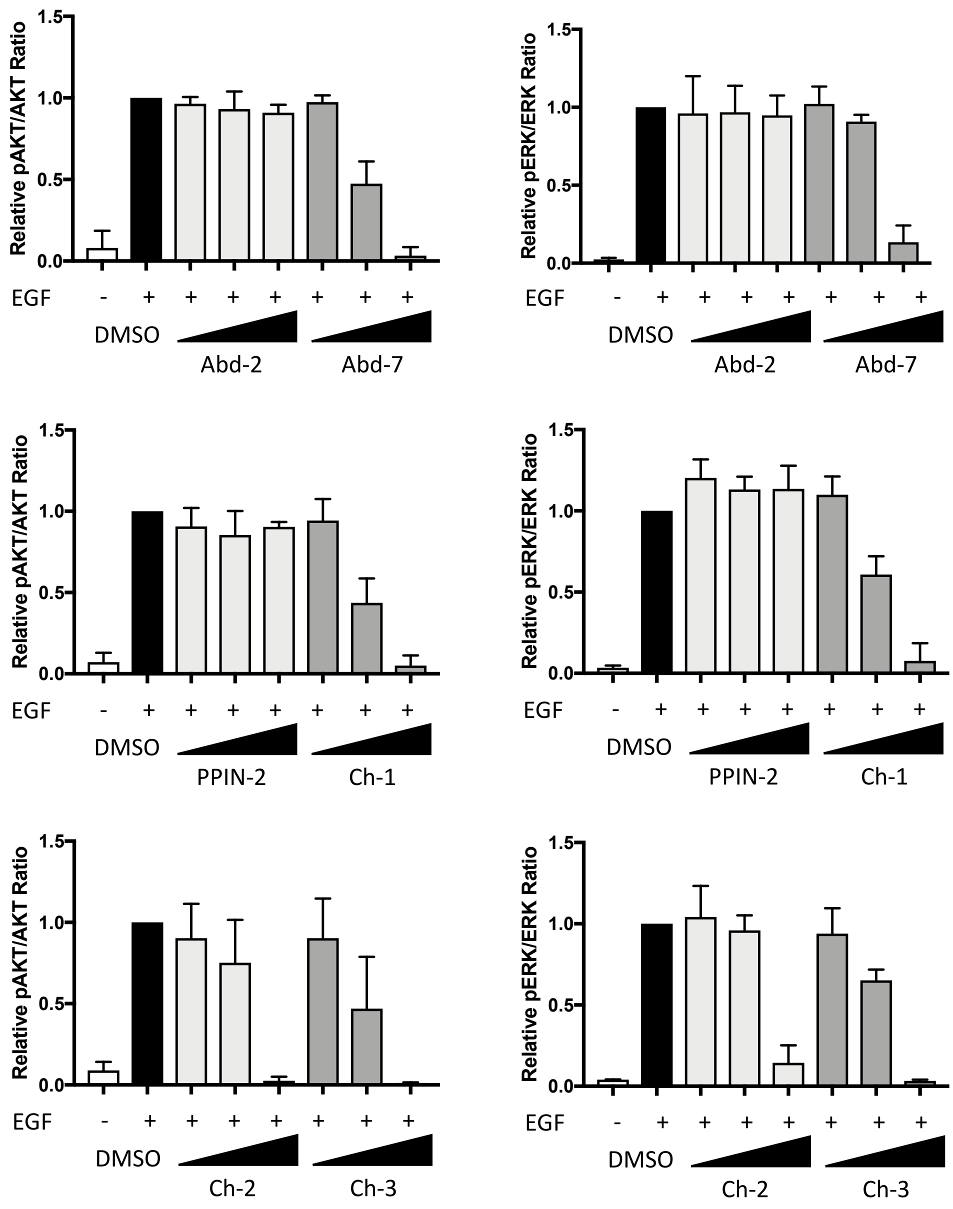

Figure S5: Quantification of data from Western blot data shown in Figure 5

Western blot data were obtained as described in Figure 5. Data were obtained from three independent experiments (biological replicates) and error bars are shown for standard deviations. Images were scanned and quantified using ImageJ software, and the relative quantity of phosphorylated protein was calculated with reference to unphosphorylated protein (eg. pAKT/AKT). The ratios were normalised against the data for DMSO-only control levels.

Compound 1

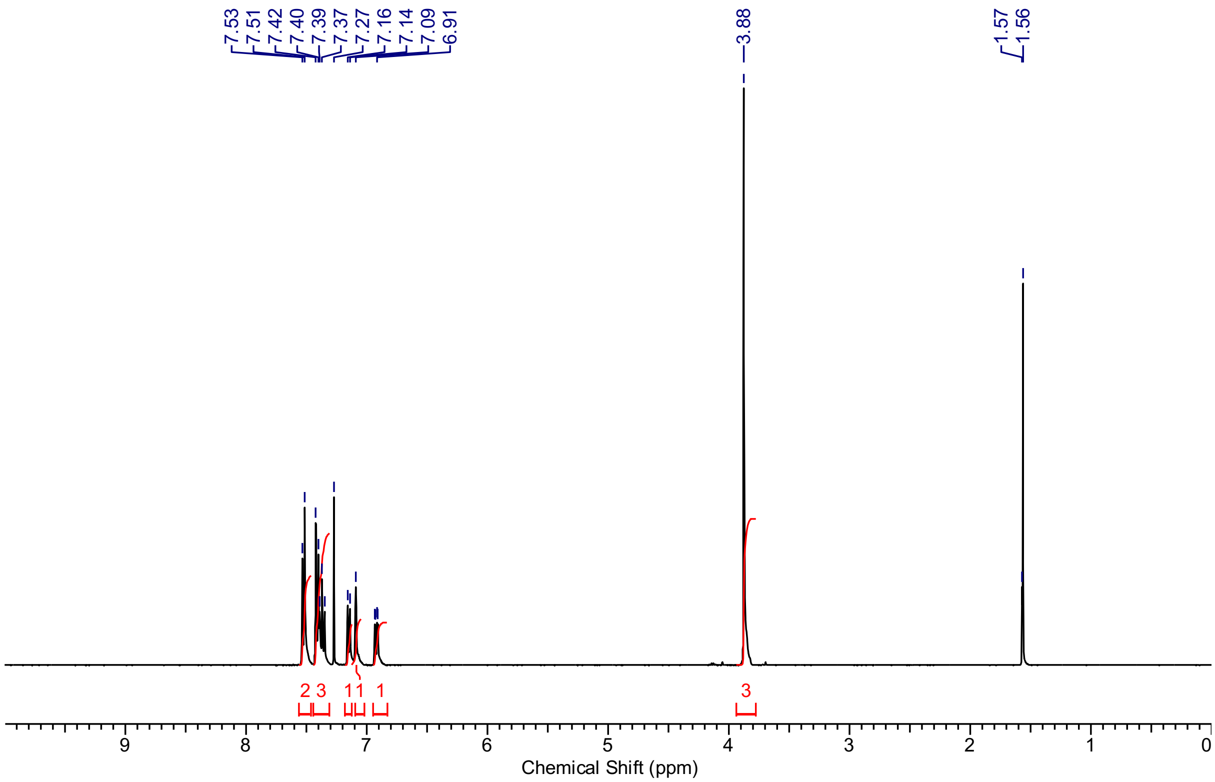
7.53
7.51
7.42
7.40
7.39
7.37
7.27
7.16
7.14
7.09
6.91
3.88
1.57
1.56
2
3
1
1
1
3
9
8
7
6
5
4
3
2
1
0
Chemical Shift (ppm)

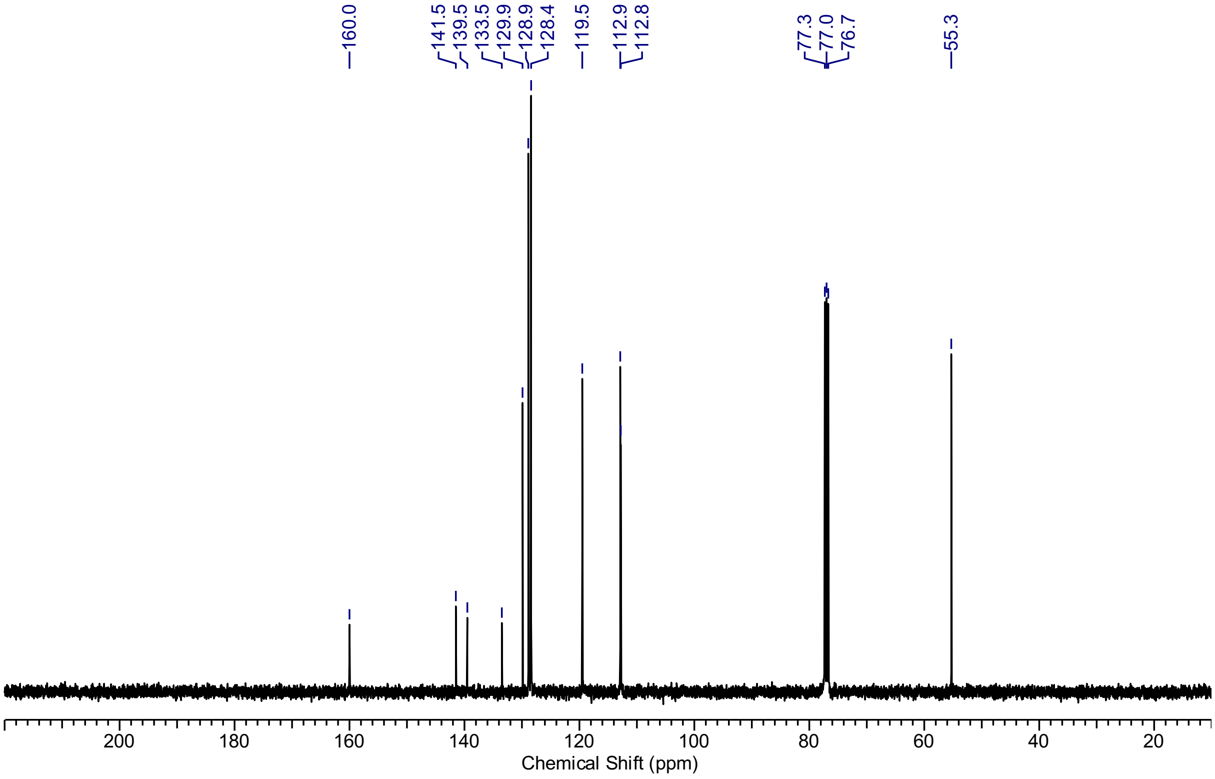
160.0
141.5
139.5
133.5
129.9
128.9
128.4
119.5
112.9
112.8
77.3
77.0
76.7
55.3
200
180
160
140
120
100
80
60
40
20
Chemical Shift (ppm)

Compound Ch-1

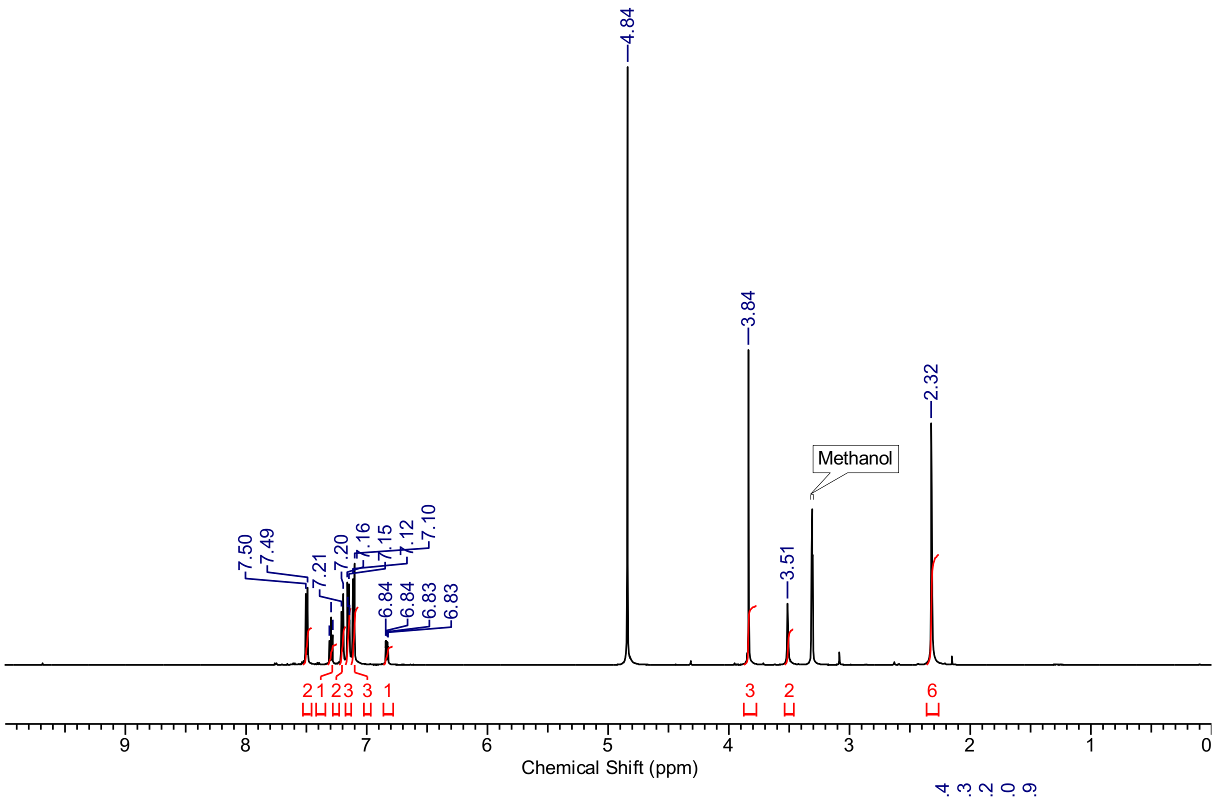

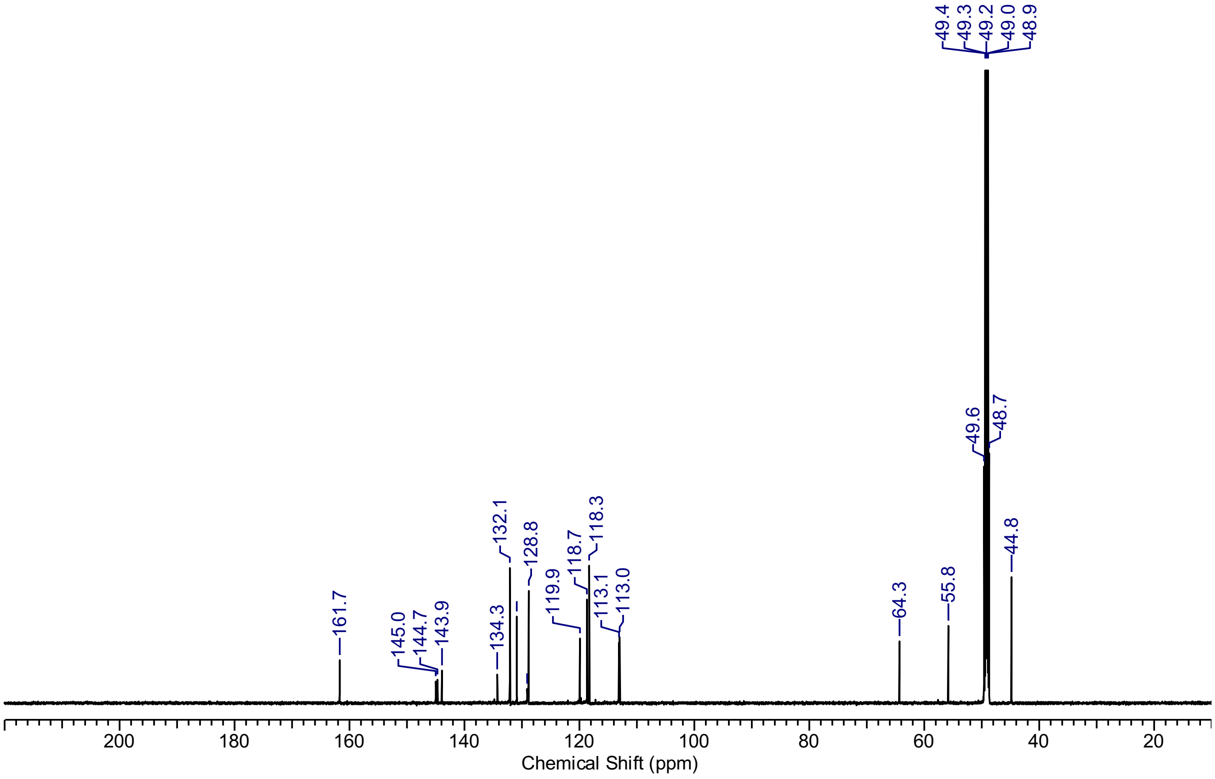

Compound 2

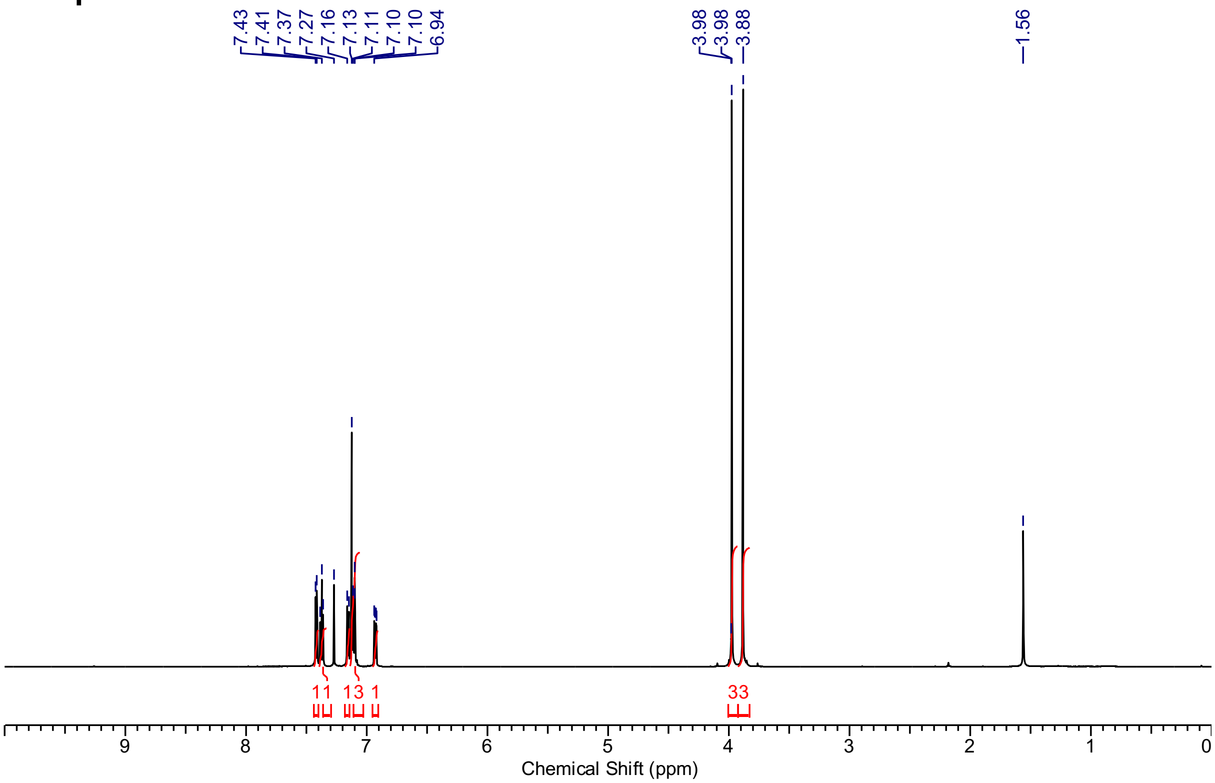
7.43
7.41
7.37
7.27
7.16
7.13
7.11
7.10
7.10
6.94
3.98
3.98
3.88
1.56
1 1 1 3 1
3 3
9 8 7 6 5 4 3 2 1 0
Chemical Shift (ppm)

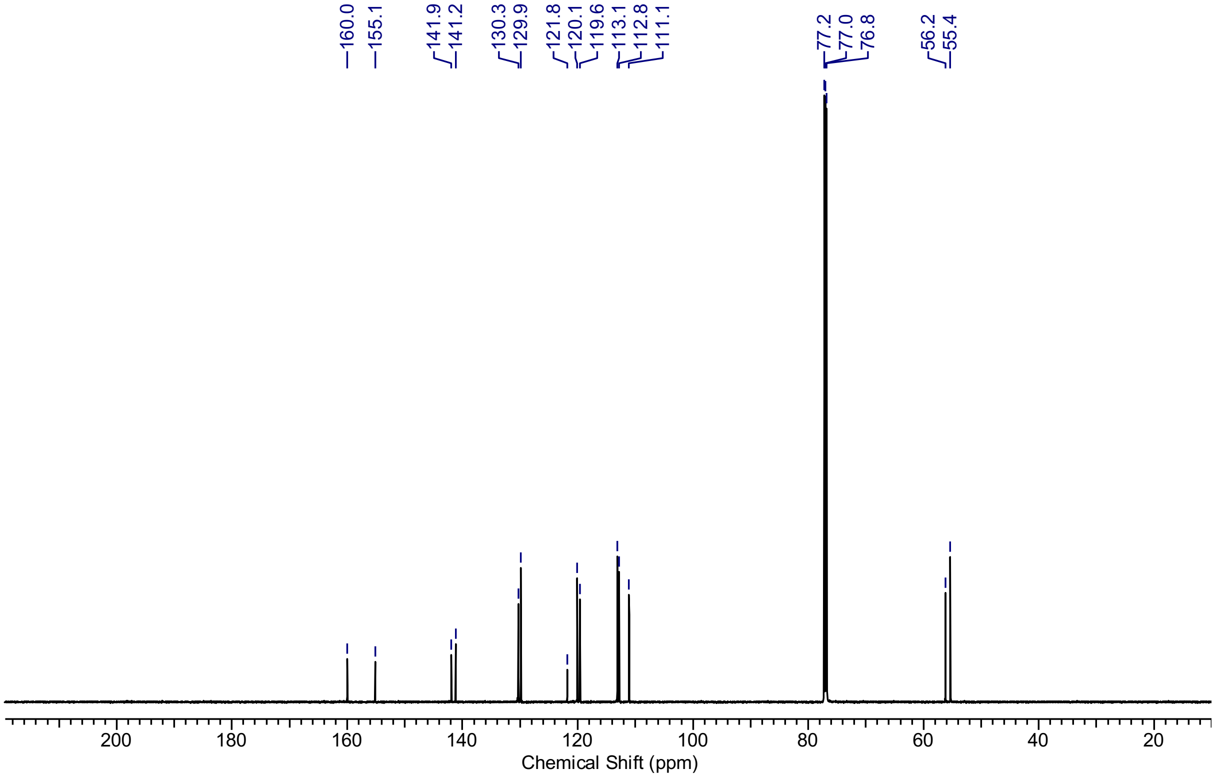
160.0
155.1
141.9
141.2
130.3
129.9
121.8
120.1
119.6
113.1
112.8
111.1
77.2
77.0
76.8
56.2
55.4
200 180 160 140 120 100 80 60 40 20
Chemical Shift (ppm)

Compound Ch-2

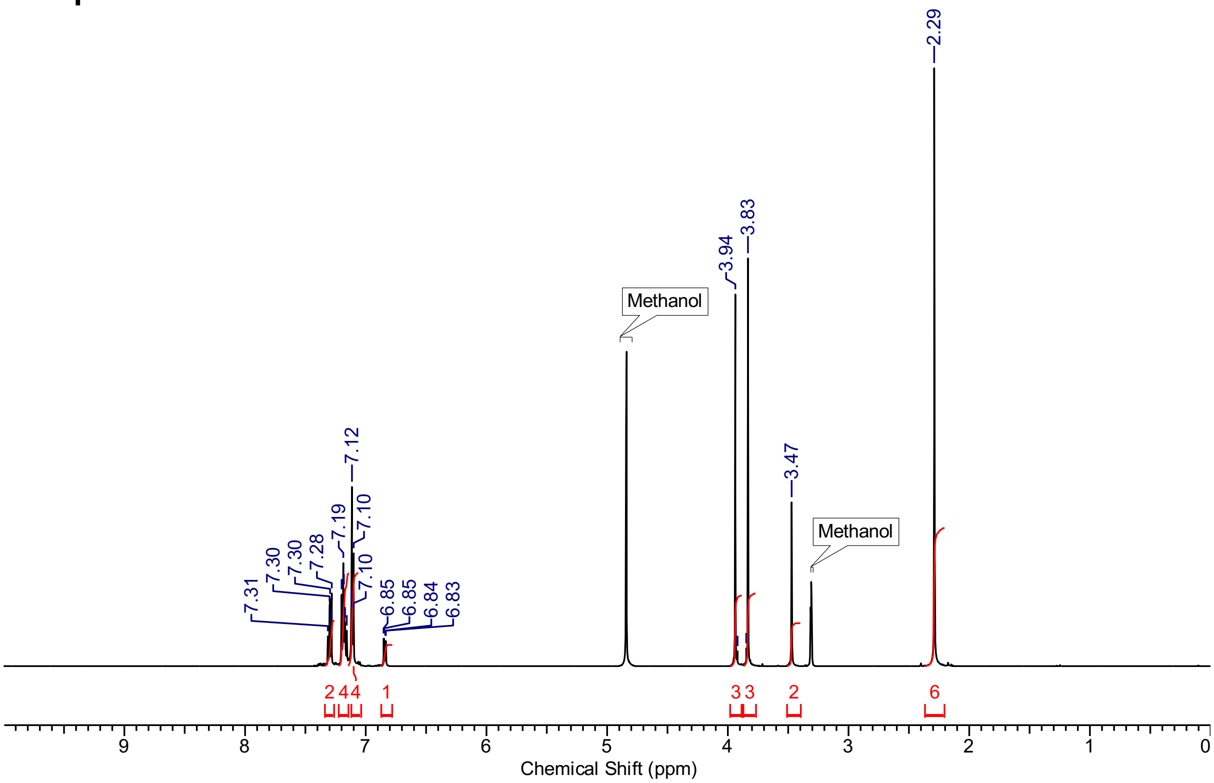

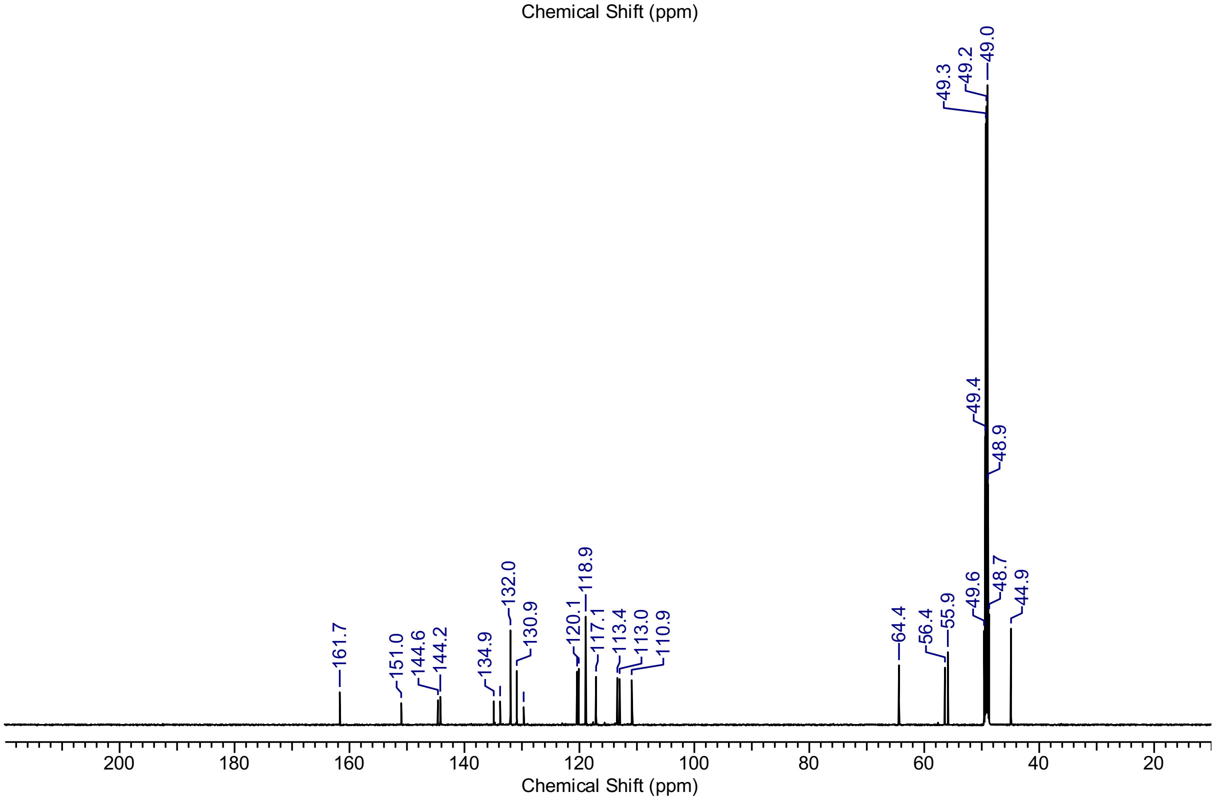

Compound 3

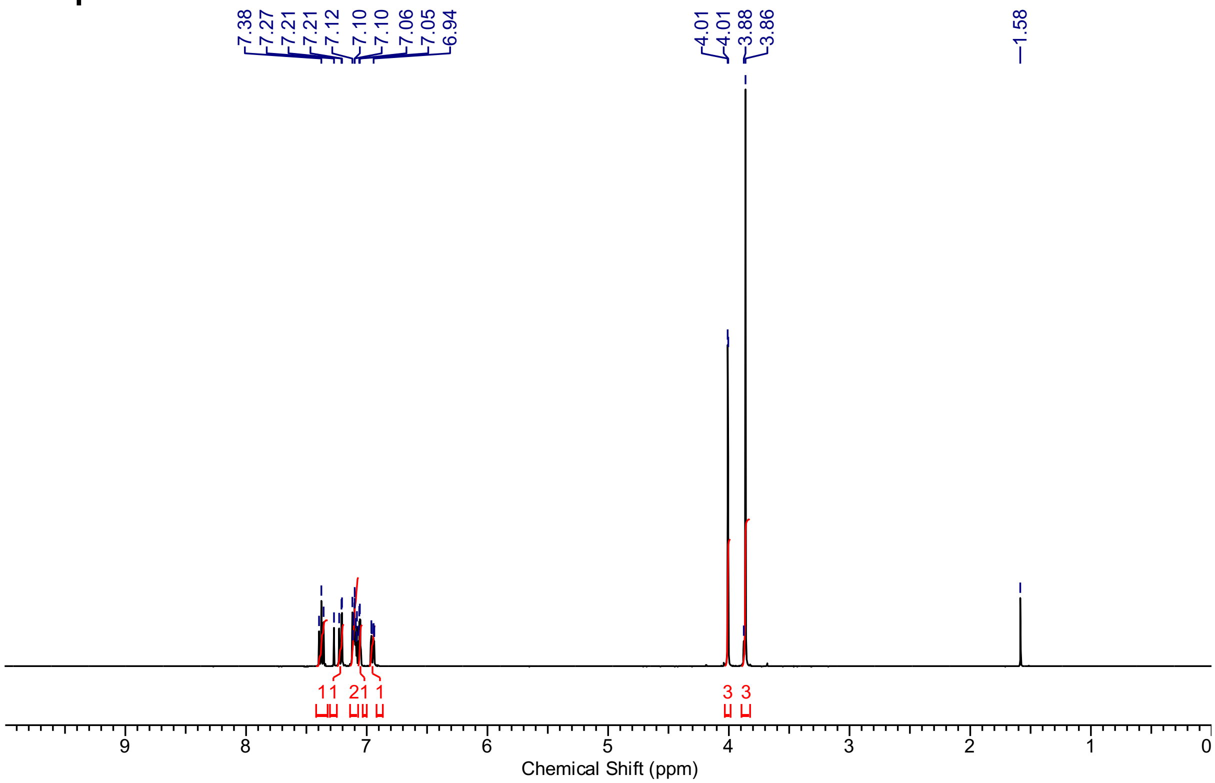
7.38
7.27
7.21
7.21
7.12
7.10
7.10
7.06
7.05
6.94
4.01
4.01
3.88
3.86
1.58
1
1
2
1
1
3
3
9
8
7
6
5
4
3
2
1
0
Chemical Shift (ppm)

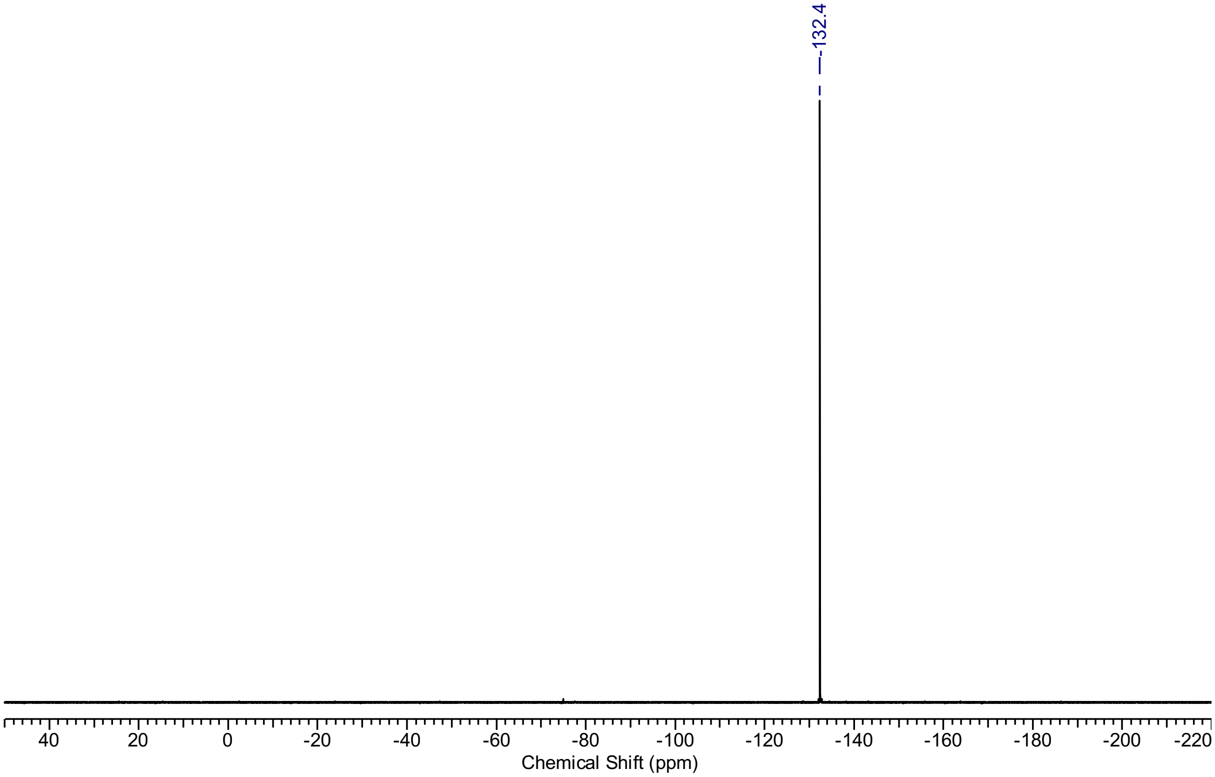
-132.4
40
20
0
-20
-40
-60
-80
-100
-120
-140
-160
-180
-200
-220
Chemical Shift (ppm)

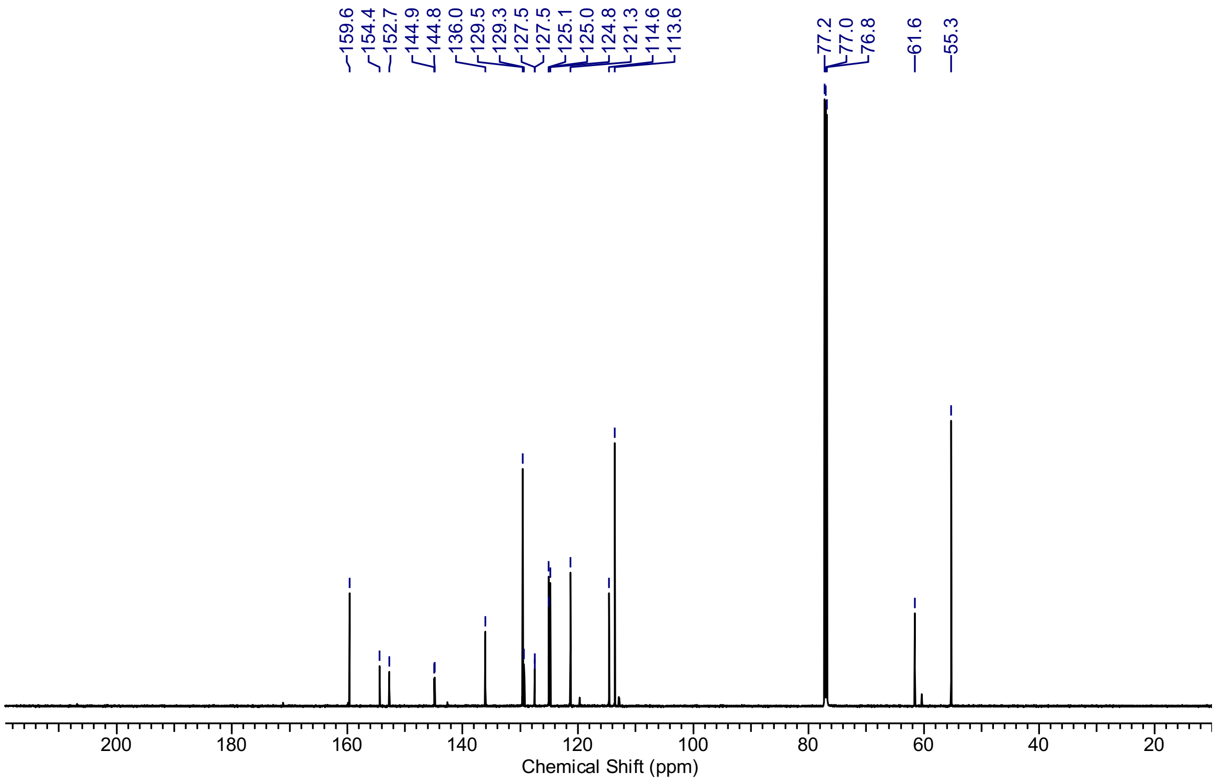

159.6
154.4
152.7
144.9
144.8
136.0
129.5
129.3
127.5
127.5
125.1
125.0
124.8
121.3
114.6
113.6
77.2
77.0
76.8
61.6
55.3
200
180
160
140
120
100
80
60
40
20
Chemical Shift (ppm)

Compound Ch-3

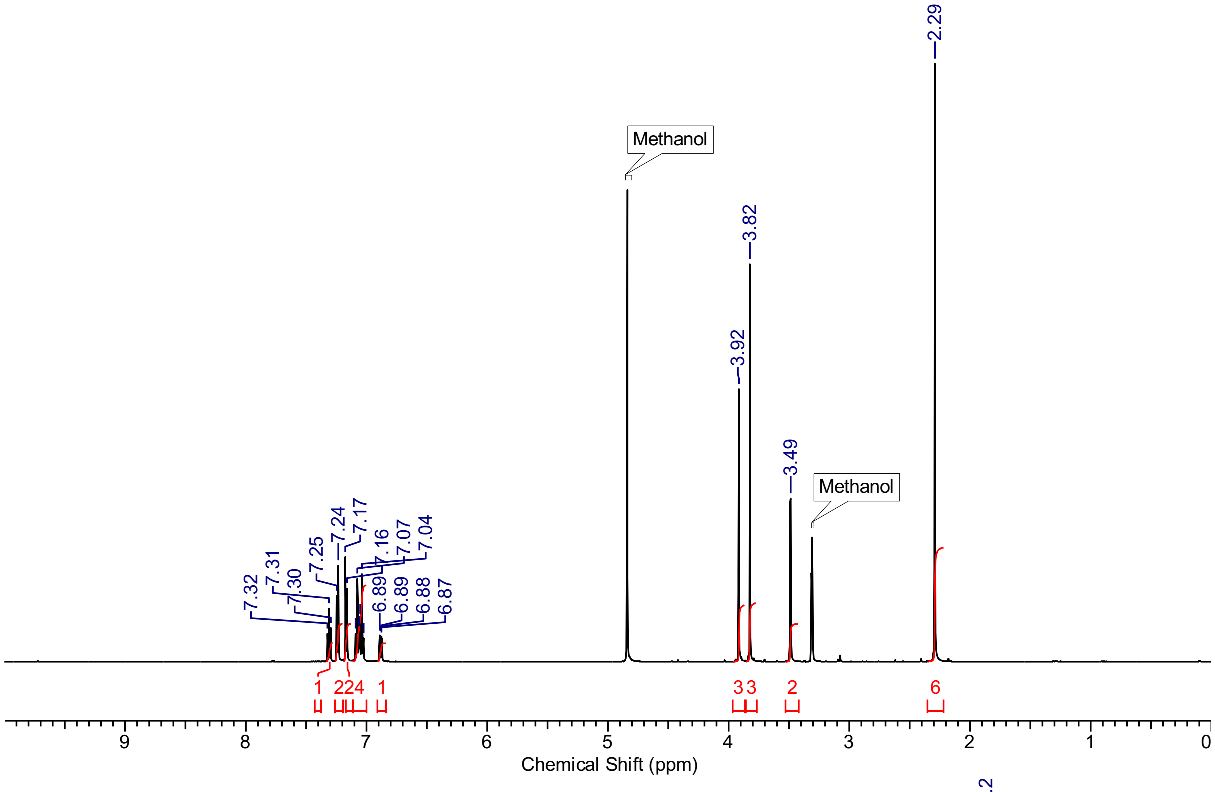

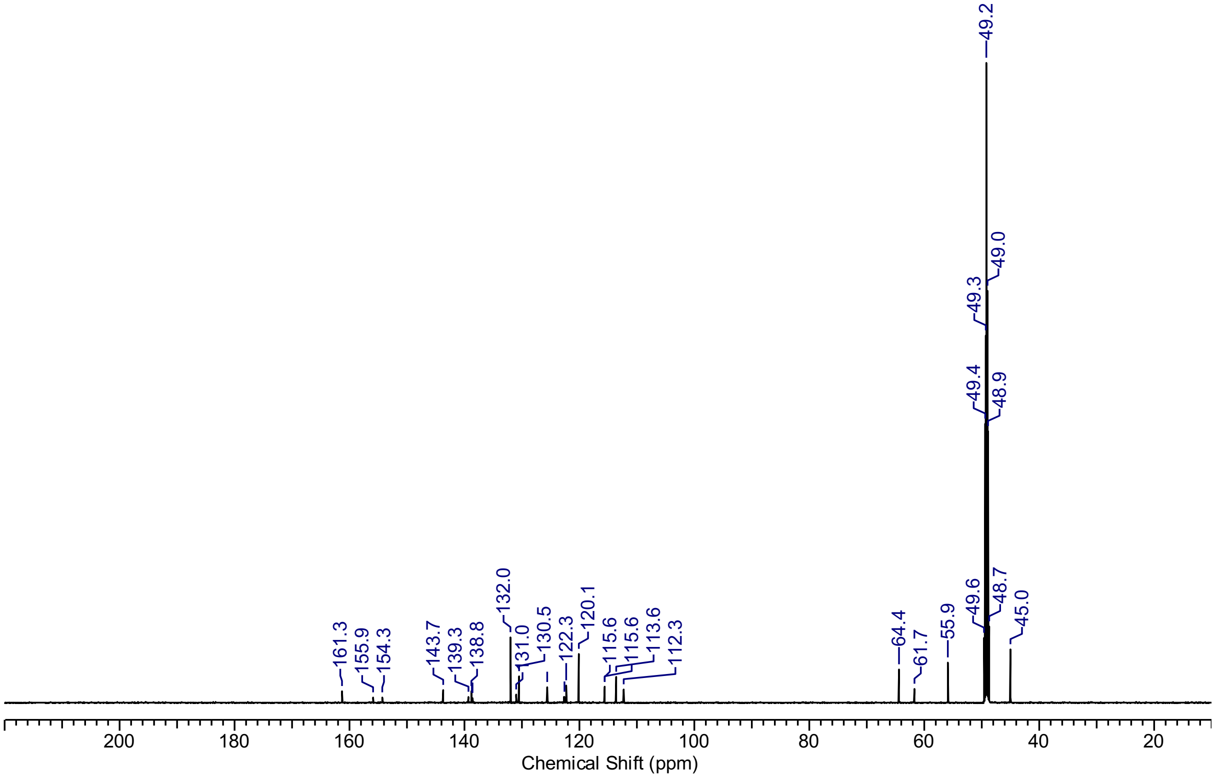

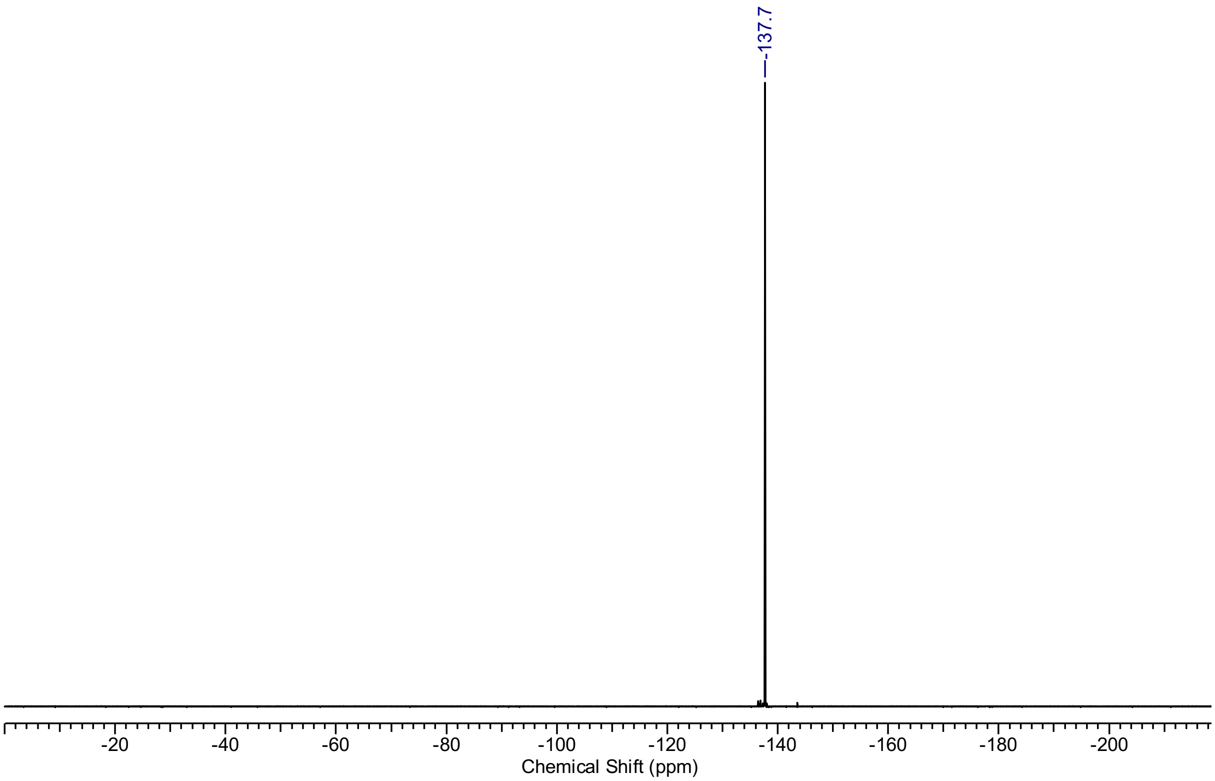

Figure S6. ^{1}H NMR and ^{13}C NMR spectra for all compounds and ^{19}F NMR for compound 3 and Ch-3

NMR spectra were recorded on Bruker Avance spectrometers (AVII400, AVIII400 or AVIIIHD600) in the deuterated solvent stated. The field was locked by external referencing to the relevant deuteron resonance. Chemical shifts (δ) are reported in parts per million (ppm) referenced to the solvent peak.

Table S1. Data processing and refinement statistics for KRAS variant Q61H with bound GTP analogue GPPNHP.

**Structure**	**$KRAS_{169}^{Q61H}$ GppNHp**
**Data Collection**	
PDB ID	6GOG
Diffraction source	Beamline i04, DLS
Temperature (K)	100
Wavelength (Å)	0.9795
Space group	$P\ 2_12_12_1$
Molecules/asymmetric unit	6
**Unit cell dimensions**	
a, b, c (Å)	63.60,118.73,156.57
α, β, γ (°)	90, 90, 90
Resolution range (Å)	94.60 - 2.05 (2.09 - 2.05)
Total No. of reflections	805,732 (24,815)
Unique reflections	74,311 (4,062)
Completeness (%)	98.8 (89.7)
Multiplicity	10.8 (6.1)
Rmeas(I)‡ (%)	18.1 (42.9)
Rmerge‡ (%)	14.9 (33.8)
Rpim(I)$ (%)	7.0 (20.6)
I/sigma	23.7 (4.3)
$CC_{1/2}$	0.994 (0.888)
**Refinement**	
No. of reflections, working set	70,508 (4,677)
No. of reflections, test set	3,728 (226)
Rwork/Rfree (%)	17.1/19.
**No. of atoms**	
Protein	8368
Water	715
**Average B factors (Å^2)**	
Protein	32.26
Ligand	26.32
Solvent	41.66
**RMSD**	
Bond lengths (Å)	0.012
Bond angles (°)	1.57

**Ramachandran plot**	
Favoured regions (%)	97.5
Additionally allowed (%)	2.5
Outliers	0.0
**MolProbity statistics**	
Molprobity score	1.24
Clash score	3.57
Rotamer outliers (%)	0.56

Values in parentheses are for data in the highest resolution shell.

Table S2. Data processing and refinement statistics for full length KRAS structures

**Structure**	**$KRAS_{188}^{G12V}$ GppNHp**	**$KRAS_{188}^{G12D}$ GppNHp**	**$KRAS_{188}^{WT}$ GppNHp**
**Data Collection**			
PDB ID	6GOE	6GOF	6GOD
Diffraction source	Beamline i04-1, DLS	Beamline i02, DLS	ID30A-1, ESRF
Temperature (K)	100	100	100
Wavelength (Å)	0.928	0.979	0.966
Space group	H3	H3	H3
Molecules/asymmetric unit	1	1	1
**Unit cell dimensions**			
a, b, c (Å)	79.88, 79.88, 78.79	78.63, 78.63, 77.54	78.76,78.76,77.18
α, β, γ (°)	90, 90, 120	90, 90, 120	90, 90, 120
Resolution range (Å)	39.94-1.60 (1.64-1.60)	39.31-1.98 (2.03-1.98)	51.11-1.70(1.73-1.70)
Total No. of reflections	126628(9332)	126182 (8935)	57807(3104)
Unique reflections	24651(1820)	12435 (900)	18963 (965)
Completeness (%)	99.7 (99.8)	99.9 (100.0)	98.0 (99.6)
Multiplicity	5.1 (5.1)	10.1 (9.9)	3.0 (3.2)
Rmeas(I)[‡] (%)	0.098(1.196)	0.171 (1.480)	0.062(0.566)
Rmerge[‡] (%)	0.088(1.072)	0.152 (1.324)	0.051 (0.468)
Rpim(I)[§] (%)	0.043 (0.526)	0.054 (0.468)	0.034 (0.311)
I/sigma	9.4(1.5)	11.2 (1.7)	11.1 (2.3)
$CC_{1/2}$	0.996 (0.528)	0.997 (0.555)	0.998(0.704)
**Refinement**			
No. of reflections, working set	24651 (2450)	12434 (1225)	18914 (1899)
No. of reflections, test set	1188 (112)	584 (72)	988 (115)
Rwork/Rfree (%)	0.183/0.220	0.181/ 0.249	0.181/ 0.236
**No. of atoms**			
Protein	1332	1382	1384
Water	112	117	101
**Average B factors (Å^2)**			
Protein	31.27	38.54	34.07
Ligand	21.44	26.90	26.91

Solvent	38.14	39.86	42.11
**RMSD**			
Bond lengths (Å)	0.023	0.013	0.020
Bond angles (°)	2.09	1.56	2.02
**Ramachandran plot**			
Favoured regions (%)	98.2	98.2	97.1
Additionally allowed (%)	1.8	1.8	2.9
Outliers	0.00	0.00	0.00
**MolProbity statistics**			
Molprobity score	1.09	1.08	1.51
Clash score	0.37	2.15	2.49
Rotamer outliers (%)	4.03	1.30	2.60

Values in parentheses are for data in the highest resolution shell.

Table S3. Data processing and refinement statistics for binding of compounds PPIN-1 and PPIN-2 to $KRAS_{169}^{Q61H}$

**Structure**	**$KRAS_{169}^{Q61H}$ + PPIN-1**	**$KRAS_{169}^{Q61H}$ + PPIN2**
**Data Collection**		
PDB ID	6GOM	6GQT
Diffraction source	Beamline i04-1, DLS	Beamline i04-1, DLS
Wavelength (Å)	0.9282	0.9282
Space group	$P2_12_12_1$	$P2_12_12_1$
Molecules/asymmetric unit	6	6
**Unit cell dimensions**		
a, b, c (Å)	64.18, 119.84, 158.60	64.27, 119.55, 158.59
α, β, γ (°)	90, 90, 90	90, 90, 90
Resolution range (Å)	66.13-1.63 (1.67-1.63)	59.8-1.69 (1.73-1.69)
Total No. of reflections	1,896,813 (67,739)	1,746,677 (70,866)
Unique reflections	152,914 (7,534)	137,251 (6764)
Completeness (%)	100 (99.3)	100 (100)
Multiplicity	12.4 (9.0)	12.7 (10.5)
Rmeas(I) ǂ (%)	8.8 (186)	11 (215)
Rmerge‡ (%)	8.8 (176)	10.6 (204)
Rpim(I) § (%)	2.5 (61.6)	3.1 (66.4)
I/sigma	15.9 (1.1)	15.3 (1.2)
$CC_{1/2}$	1 (0.5)	1 (0.6)
**Refinement**		
No.of reflections, working set	145,381	130,344
No. of reflections, test set	7428	6817
Rwork/Rfree (%)	18.6/20.7	18.8/21.1
**No. of atoms**		
Protein	8100	8144
Water	776	814
**Average B factors (Å2)**		
Protein	34.9	36.127
Ligand	38.2	98.2
Water	42.2	43.2
**RMSD**		
Bond lengths (Å)	0.02	0.02
Bond angles (°)	1.98	1.91
**Ramachandran plot**		

Favoured regions (%)	97.9	97.6
Additionally allowed (%)	2.1	2.4
Outliers	0	0
**MolProbity statistics**		
Molprobity score	0.94	1.22
Clash score	1.75	2.56
Rotamer outliers (%)	0.94	1.4

Values in parentheses are for data in the highest resolution shell.

Table S4. Data processing and refinement statistics for binding of compounds Ch-1, Ch-2 and Ch-3 to $KRAS^{Q61H}$

Structure	$KRAS_{169}^{Q61H}$ + Ch-1	$KRAS_{169}^{Q61H}$ + Ch-2	$KRAS_{169}^{Q61H}$ + Ch-3
**Data Collection**			
PDB ID	6GQW	6GQX	6GQY
Diffraction source	Beamline P14, DESY	Beamline P14, DESY	Beamline P14, DESY
Wavelength (Å)	0.9763	0.9763	0.9763
Space group	$P2_12_12_1$	$P2_12_12_1$	$P2_12_12_1$
Molecules/asymmetric unit	6	6	6
**Unit cell dimensions**			
a, b, c (Å)	63.48, 118.54, 156.53	63.19, 118.03, 156.37	63.53, 118.68, 157.00
α, β, γ (°)	90, 90, 90	90, 90, 90	90, 90, 90
Resolution range (Å)	94.5-2.8 (2.95-2.8)	156-2.2 (2.26-2.20)	118-2.75 (2.88-2.75)
Total No. of reflections	202,993 (29,729)	577,336 (38,536)	332,585 (45,297)
Unique reflections	27,994 (4,121)	60,229 (4,369)	31,557 (4,134)
Completeness (%)	92.9 (95.8)	100 (99.8)	99.8 (99.9)
Multiplicity	7.3 (7.2)	9.6 (8.8)	10.5 (11.0)
Rmeas(I) ǂ (%)	16.9 (96.1)	10.8 (93.9)	15.9 (59.1)
Rmerge‡ (%)	14.6 (83.3)	9.7 (83.0)	14.3 (53.5)
Rpim(I) § (%)	8.4(47.5)	4.7 (43.2)	6.7 (24.5)
I/sigma	12.3 (3.7)	15.6 (3.3)	16.0 (4.2)
$CC_{1/2}$	0.99 (0.83)	0.998 (0.908)	0.997 (0.916)
**Refinement**			
No.of reflections, working set	26,537	57,101	29,946
No. of reflections, test set	1,403	3,016	1,561
Rwork/Rfree (%)	18.9/22.4	20.3/23.0	20.5/24.7
**No. of atoms**			
Protein	8319	8202	8289
Water	36	95	33
**Average B factors**			
Protein	54.6	43.6	46.3
Ligand	33.6	47.5	60.0
Water	78.5	31.7	33.5
**RMSD**			
Bond lengths (Å)	0.01	0.01	0.01
Bond angles (°)	1.36	1.45	1.5
**Ramachandran plot**			

Favoured regions (%)	97.3	97.5	96.6
Additionally allowed (%)	2.7	2.5	3.4
Outliers	0	0	0
**MolProbity statistics**			
Molprobity score	1.37	1.24	1.42
Clash score	2.77	2.95	2.34
Rotamer outliers (%)	1.7	1.2	1.7

Values in parentheses are for data in the highest resolution shell.

Table S5. Compound properties for comparison

	Abd-7	PPN-1	PPN-2	Ch-1	Ch-2	Ch-3
**L.E.**	0.34	0.30	0.27	N/A	0.35	N/A
**Cell Viability (μM, 48 h)**	10.8	Inactive	Inactive	9.5	9.9	7.8
**Cell Viability (μM, 72 h)**	8.2	Inactive	Inactive	5.3	7.2	4.5

References

1. Hermann C, Horn G, Spaargaren M, & Wittinghofer A (1996) Differential interaction of the ras family GTP-binding proteins H-RAs, Rap1A, and R-Ras with the putative effector molecules Raf kinase and Ral-guanine nucleotide exchange factor. *J Biol Chem* 271:6794-6800.
2. Luft JR & DeTitta GT (1999) A method to produce microseed stock for use in the crystallization of biological macromolecules. *Acta Crystallogr D Biol Crystallogr* 55(Pt 5):988-993.
3. McCoy AJ*, et al.* (2007) Phaser crystallographic software. *J Appl Crystallogr* 40(Pt 4):658-674.
4. Murshudov GN, Vagin AA, & Dodson EJ (1997) Refinement of macromolecular structures by the maximum-likelihood method. *Acta Crystallogr D Biol Crystallogr* 53(Pt 3):240-255.
5. Emsley P, Lohkamp B, Scott WG, & Cowtan K (2010) Features and development of Coot. *Acta Crystallogr D Biol Crystallogr* 66(Pt 4):486-501.
6. Chen VB*, et al.* (2010) MolProbity: all-atom structure validation for macromolecular crystallography. *Acta Crystallogr D Biol Crystallogr* 66(Pt 1):12-21.
7. Laskowski RA, MacArthur MW, Moss DS, & Thornton JM (1993) PROCHECK - a program to check the stereochemical quality of protein stuctures. *J. App. Cryst.* 26(283-291).
8. Adams PD*, et al.* (2010) PHENIX: a comprehensive Python-based system for macromolecular structure solution. *Acta Crystallogr D Biol Crystallogr* 66(Pt 2):213-221.
9. Dalvit C, Fogliatto G, Stewart A, Veronesi M, & Stockman B (2001) WaterLOGSY as a method for primary NMR screening: practical aspects and range of applicability. *J Biomol NMR* 21(4):349-359.
10. Cheeseright T, Mackey M, Rose S, & Vinter A (2006) Molecular Field Extrema as Descriptors of Biological Activity: Definition and Validation. *J. Chem. Inf. Model.* 46:665-676.
11. Bery N*, et al.* (2018) BRET-based RAS biosensors that show a novel small molecule is an inhibitor of RAS-effector protein-protein interactions. *Elife* 7. doi.org/10.7554/eLife.37122
